# Glutathione Transferases Superfamily: Cold-Inducible Expression of Distinct *GST* Genes in *Brassica oleracea*

**DOI:** 10.3390/ijms17081211

**Published:** 2016-07-27

**Authors:** Harshavardhanan Vijayakumar, Senthil Kumar Thamilarasan, Ashokraj Shanmugam, Sathishkumar Natarajan, Hee-Jeong Jung, Jong-In Park, HyeRan Kim, Mi-Young Chung, Ill-Sup Nou

**Affiliations:** 1 Department of Horticulture, Sunchon National University, 255, Jungang-ro, Suncheon 57922, Korea; vharshavardhanan@gmail.com (H.V.); seninfobio@gmail.com (S.K.T.); araj866@gmail.com (A.S.); sathisbioinfo@gmail.com (S.N.); my-656@hanmail.net (H.-J.J.); jipark@sunchon.ac.kr (J.-I.P.); 2 Plant Systems Engineering Center, Korea Research Institute of Bioscience and Biotechnology (KRIBB), 125 Gwahangno, Daejeon 34141, Korea; kimhr@kribb.re.kr; 3 Department of Agricultural Education, Sunchon National University, 255, Jungang-ro, Suncheon 57922, Korea; queen@sunchon.ac.kr

**Keywords:** cold, glutathione transferase (GST), *Brassica oleracea*, contrasting genotypes, tolerance

## Abstract

Plants, as sessile organisms, can suffer serious growth and developmental consequences under cold stress conditions. Glutathione transferases (GSTs, EC 2.5.1.18) are ubiquitous and multifunctional conjugating proteins, which play a major role in stress responses by preventing oxidative damage by reactive oxygen species (ROS). Currently, understanding of their function(s) during different biochemical and signaling pathways under cold stress condition remain unclear. In this study, using combined computational strategy, we identified 65 *Brassica oleracea* glutathione transferases (*BoGST*) and characterized them based on evolutionary analysis into 11 classes. Inter-species and intra-species duplication was evident between *BoGST*s and *Arabidopsis* GSTs. Based on localization analyses, we propose possible pathways in which *GST* genes are involved during cold stress. Further, expression analysis of the predicted putative functions for *GST* genes were investigated in two cold contrasting genotypes (cold tolerance and susceptible) under cold condition, most of these genes were highly expressed at 6 h and 1 h in the cold tolerant (CT) and cold susceptible (CS) lines, respectively. Overall, *BoGSTU19*, *BoGSTU24*, *BoGSTF10* are candidate genes highly expressed in *B. oleracea*. Further investigation of GST superfamily in *B. oleracea* will aid in understanding complex mechanism underlying cold tolerance in plants.

## 1. Introduction

Cold stress is detrimental to plant growth and development, thus affecting agricultural productivity worldwide. During low temperature conditions, cold-tolerant plants activate a tolerance mechanism called cold acclimation, which prevents ice formation within the vegetative cells. However, many agricultural crops lack this cold acclimation mechanism. In general, plants achieve tolerance to cold stress by modifying biochemical and physiological factors at the cellular and molecular level. Cold acclimation involves expression of a set of so-called cold-regulated (*COR*) genes, induction of which is mediated by effector genes and various transcription factors [1]. In addition to their basic role of protecting cells during cold stress conditions, *COR* genes regulate signal transduction of defense-related and secondary metabolite genes [2]. Cold stress responses involve cis-acting elements such as ABREs (abscisic acid response element), DREs (c-repeat/dehydration responsive elements), and LTREs (low-temperature-responsive elements), which are regulated by abscisic acid (ABA)-dependent or -independent pathways [3]. ABA and reactive oxygen species (ROS) play vital roles as second messengers via Ca^2+^ signaling, inducing *COR* genes such as *ERD6* [1], *LOS5*, *FRO1* [4], *AP2*/*ERF* [5,6], *bZIP* [7] and *CBF*s [8]. Notably, there is also an increase in secondary metabolite accumulation in the plant cell during cold stress. Cold conditions induce increased accumulation of sugar [9], polyamines [10], anthocyanins [11], and glucosinolates [12]. Increased activity of enzymatic (superoxide dismutase (SOD), GST, glutathione reductase (GR), and glutathione peroxidases (GPX)) and non-enzymatic antioxidants (GSH, tripeptide thiols, and vitamins) are also evident in cold-stressed tissues [13]. Additionally, post-translational modifications, such as sumoylation (conjugation of small ubiquitin-like modifier (SUMO) proteins to target proteins) carried out by *SIZ1* gene products during stress, play roles in cold tolerance [14]. *SIZ1* regulates the expression of *CBF*/*DREB* genes by inhibiting *MYB* genes, thereby enhancing low temperature tolerance [15].

Plant glutathione transferases (GSTs, EC 2.5.1.18) represent a complex superfamily of proteins with multiple roles, involving site-specific (G-site) tripeptide conjugation (glutathione, GSH, and γ-Glu-Cys-Gly) leading to reduction of a wide range of electrophilic and hydrophobic substrates and redox buffering. GSTs play a vital role in glucosinolate biosynthesis and metabolism [16], transport of anthocyanin [17,18], and xenobiotic metabolism [19]. Thus far, evolutionary analysis of GSTs found in eukaryote photosynthetic organisms elucidated 14 major classes in this superfamily [20,21]. Currently, in plants, GSTs are grouped into tau, phi, theta, zeta, lambda, DHAR (dehydroascorbate reductase), EF1G (elongation factor 1 γ), TCHQD (tetrachlorohydroquinone dehalogenase), GHR (glutathionyl hydroquinone reductase), GST2N (glutathione transferases with two thioredoxin), and mPGES2 (microsomal prostaglandin e synthase type 2) classes based on sequence similarity, immunological reactivity, kinetic properties, and structural conformation. Among these classes, tau, phi, DHAR, and lambda GSTs are plant-specific proteins. GST proteins in all phyla typically contain GST N-domain (thioredoxin-like domain, conjugation of GSH moiety, G-site) and GST C-domain (bind to hydrophobic substrates, H-site) except GST2N class, which contains two repeated GST N-domain and lack terminal GST C-domain. A serine residue in the N-terminal region acts as the active site of GST proteins [22], except in those of the lambda, DHAR, GHR, and mPGES2 classes wherein the serine is replaced with a cysteine residue in their active site [21,23]. GSTs were originally considered to serve mainly for xenobiotic detoxification until the discovery of their functions in preventing oxidative damage to cells due to biotic and abiotic stresses such as pathogen infection, ROS, UV radiation, and heavy metal toxicity [24,25,26]. Dixon et al. (2009) reported that tau and phi class GSTs have a wide range of substrate specificity in *Arabidopsis*, which is correlated with high tolerance against cold, dehydration, UV, oxidative stress, salt, and heavy metals [27]. In *Arabidopsis*, phi class glutathione transferases (GSTF2) has high affinity to heterocyclic compounds and aids in intracellular transport of defense-related genes [28]. DHAR maintains a reduced ascorbic acid pool within the cell, acting as an antioxidant protein [29], and shows up-regulation during abiotic stresses such as light and drought [21]. Further, lambda *GST* genes show increased expression under heavy metal stress compared to other *GST* genes [30]. Among the GST superfamily, few tau, phi and theta GSTs have been known for high glutathione peroxidase (GPOX) activity [22,31], and *Euphorbia esula* GSTT1 exhibits up-regulation during drought stress and treatment with xenobiotics [32]. Zeta GSTs are known for their roles in tyrosine catabolism [33] and aid during the germination stage under chilling and salt stress in *Euphorbia esula* [32]. Little information is interpreted related to GHR and mPGES2 classes roles during environmental conditions in plants, however microarray data of *Arabidopsis* show differential regulation during various abiotic stress [21].

*Brassica oleracea* includes many important crops in the Brassicaceae family, namely cabbage (*B. oleracea* var. *capitata*), broccoli (*B. oleracea* var. *italica*), cauliflower (*B. oleracea* var. *botrytis*), kale (*B. oleracea* var. *acephala*), Brussels sprouts (*B. oleracea* var. *gemmifera*), collard greens (*B. oleracea* var. *acephala*), kohlrabi (*B. oleracea* var. *gongylodes*), and Chinese broccoli (*B. oleracea* var. *alboglabra*). Most *Brassica* species contain high levels of proteins and diversified secondary metabolites [16] that have unique phytochemical characteristics including anti-carcinogenic properties in humans [34]. Cabbage and broccoli are the major agricultural crops of *B. oleracea*. In this study, a combined computational strategy comprising HMM (hidden Markov model) profile scan coupled with BLAST analysis of the sequenced *B. oleracea* genome data from *Brassica* databases was employed to identify *GST* genes, and further raw data were processed and screened for candidate genes based on coding and protein sequences. We identified and characterized 65 *BoGST* genes. Moreover, genome-wide expression analysis was performed in two contrasting inbred lines of *B. oleracea*, *Bo*106 (cold tolerant (CT)) and *Bo*107 (cold susceptible (CS)), during cold treatment (4 °C) to reveal possible candidate gene involved during cold tolerance.

## 2. Results and Discussion

### 2.1. Identification of GST Genes in B. oleracea

The release of the draft genome of *B. oleracea* to public databases (Brad, Bolbase and EnsemblPlants) has made it possible to analyze gene families based on annotation from the *Arabidopsis* genome. Here, we identified a comprehensive set of *GST* genes from the *B. oleracea* genome using combined computational analysis comprising HMM profiling and BLAST analyses. A series of systematic analyses was performed to assemble the final set of protein sequence. Firstly, *Arabidopsis* GST proteins (55 proteins) were collected using locus IDs published in Dixon and Edwards 2010. Based on the 3D structure of GST proteins in *Arabidopsis*, Armstrong [35] reported that GST N- and C-domains are the basic domain constituents of GST proteins. To spot the functional domains of those sequences, domain analysis was carried out and domain sequences of those proteins were used as input to build a GST-specific HMM profile. Secondly, two peptide datasets, from Bolbase and EnsemblPlants databases, were used as queries against the GST-specific HMM profile. We obtained 107 and 1109 proteins, respectively, from the datasets. From the HMMER results, multiple hits of the same genes were inferred, signifying that the assembled draft genome of *B. oleracea* contains multiple copies or fractional copies of the same gene. Manually, we identified 89 putative annotated *GST* genes from *Brassica* databases. We evaluated all 1305 identified proteins (which includes redundancy) to identify GST genes based on domain occurrence. In total, 65 non-redundant GST proteins were identified based on the HMM results and annotation searches showing they contained only GST-specific domains. Finally, we verified the annotations of these 65 protein sequences using local BLASTP against the *Arabidopsis* genome and NCBI database using customized E values (1 × 10^−3^, 1 × 10^−10^, 1 × 10^−30^ and 1 × 10^−50^). The results from each analysis were in agreement, showing paralogous and orthologous genes among *B. oleracea* (37 proteins), *B. napus* (21 proteins), *B. rapa* (6 proteins), and *Zea mays* (1 proteins) (Table S1). Also, results obtained from GST-specific HMM profile retrieved form Pfam database were similar to that of *Arabidopsis* GST-specific HMM profile (data not shown). GSTs are studied in a range of plant species such as *Arabidopsis* [22], barley [36], soybean [37], maize [37], rice [38], tomato [39], and *Populus trichocarpa* [40], and the total numbers of *GST* genes in selected crops are summarized in Table 1. Results of BLAST searches of BoGST proteins against the *Arabidopsis* genome were similar to those for orthologous genes as annotated in the Brad database (data not shown).

Our domain analysis results confirmed the presence of both GST N- (thioredoxin-like) and GST C-domains (hydrophobic or electrophilic binding) in all GST proteins except two proteins (Bol010024 and Bol015341) which contains two repeats of thioredoxin domain and lack terminal C-domain (Table S2). From our prediction results, we also found other specific and multi domains such as EF1G (elongation factor 1 γ, pfam00647), GSTA (glutathione *S*-transferase multi-complex domain, COG0625), MaiA (maleylacetoacetate isomerase, TIGR01262) and ECM4 (glutathionyl-hydroquinone reductase, COG0435). Similarly, domain analysis of AtGST and BrGST proteins (uncharacterized) using the SMART and conserved domains database (CDD) database generated similar results (data not shown). The thioredoxin-like domain comprises βαβαββα motifs that make three layers of β-sheet enclosed with two α-helixes on either side (α/β/α) [41], and there is a small peptide sequence between the N-terminus and C-terminus called the linker region (8–15 aa) that connects these two termini [42]. Mohsenzadeh et al. (2011) reported that the C-terminal region of GST confers the substrate selectivity between the GST classes [43]. Secondary structure prediction using the garnier program employing the GOR method in the EMBOSS server showed a higher percentage of α-helix than β-sheets (Table S3). Overall, the 65 BoGST proteins have open reading frames ranging from 570–1248 bp, with predicted protein lengths between 189 and 415 amino acids, and different numbers of exons in their gene structure. The predicted molecular weights, pIs, and stability indexes ranged between 21.37–46.57 (kDa), 4.96–9.5 and 27.33–60.31, respectively. Based on their stability index values, 41 and 24 proteins were characterized as stable and unstable proteins, respectively (Table S3).

### 2.2. Classification and Sequence Characterization of BoGST Genes

**Figure 1:**
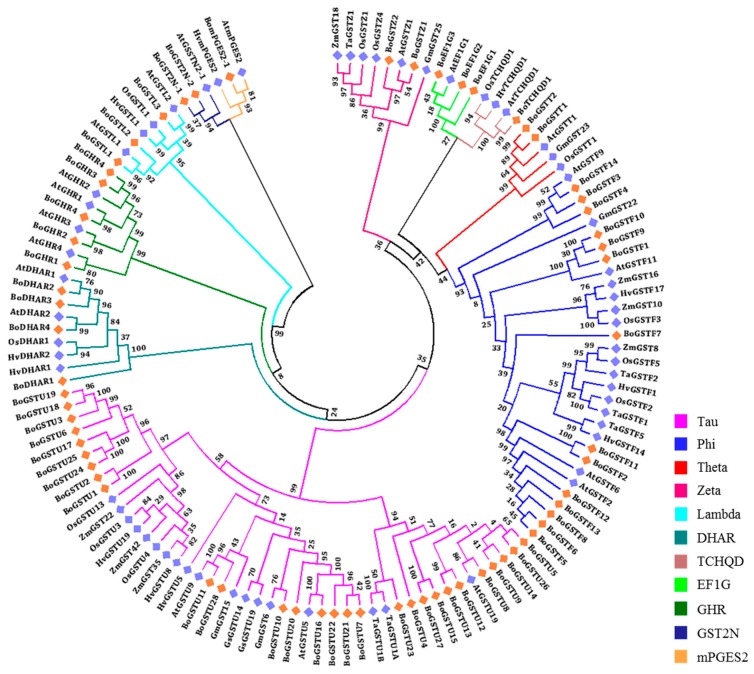
Phylogenetic trees and classification of glutathione transferase (GST) proteins of *B. oleracea* using *Arabidopsis*, rice, barley, wheat, sorghum, maize, and soybean published GST proteins. The unrooted phylogenetic trees was constructed based on multiple sequence alignment using ClustalW followed by maximum-likelihood method using JTT model MEGA6.06 software. Highlighted genes with diamond symbol, orange color represents BoGSTs and blue color represents orthologous *GST* genes.

To distinguish between mammalian GSTs and plant GSTs, a classification system was first proposed by Droog (1997) [45], and later modified [46] based on gene association within the genome. Finally, a unified nomenclature system for plant GSTs was adopted [22]. Plant GSTs are classified into eight major classes, namely tau (U), phi (F), theta (T), zeta (Z), lambda (L), DHAR, TCHQD, and EF1G. To investigate the evolutionary relationships of BoGST proteins, known GST proteins from GST superfamilies characterized in monocots (rice, maize, wheat, and barley) and dicots (*Arabidopsis*, soybean and wild soybean) were collected (Tables S4 and S5). Here, we classified the BoGSTs based on their evolutionary relationships with GSTs from other species. The two largest and most abundant classes, tau and phi, had 28 and 14 closely grouped BoGSTs, respectively, whereas three BoGSTs were separately placed in the lambda and EF1G classes and two BoGSTs in theta and zeta classes. Among the rest of the BoGSTs, four and one were clustered in DHAR, and TCHQD classes, respectively (Figure 1, Figure S1). An additional eight proteins (Bol001864, Bol004474, Bol012366, Bol012372, Bol024359, Bol010024, Bol015341, and Bo035968) were not placed into any of the above mentioned plant classes. Based on BLAST results and domain analysis, five proteins (Bol001864, Bol004474, Bol012366, Bol012372, and Bol024359) were annotated as glutathionyl hydroquinone reductase (GHR) and one protein (Bol035968) as microsomal prostaglandin E synthase type 2 (mPGES-2) with both GST N- and GST C-domain, so we named these classes of proteins as BoGHR and BomPGES2, respectively (data not shown). The remaining two proteins (Bol010024 and Bol015431) consist two repeats of GST N-domain and lack C-terminal, hence these two proteins were classified as GST2N class. A summary of all *GST* genes in *B. oleracea* is given in Table 2.

In plants, various classes of *GST* genes respond to cold stress in different species. Specifically, tau GST members in *Arabidopsis* [47], phi GSTs in *Solanum* species [48], theta GSTs in *Euphorbia* [32], and zeta GSTs as well as the *TCHQD* gene in *O. sativa* [49,50] show cold-responsive expression. Members of a cold-induced antioxidant enzyme family, DHAR, show activity specifically for rapid H_2_O_2_ scavenging in the chloroplast through the water-water cycle to remove H_2_O_2_ from the cell [51]. Ahsan et al. (2008) reported that GST omega class genes are closely related to human glutathione *S*-transferase omega (*GSTO*) genes and are involved in heavy metal detoxification in rice roots [52]. However, the roles of other GST classes in plants needs to be elucidated during cold stress and other abiotic stresses. Mapping of enzymes or proteins in biochemical pathways can help in understanding their biological function. *AtGSTZ1-1* shows maleylacetone isomerase (MAI) activity with a GSH-dependent reaction, involved in tyrosine metabolism in *Arabidopsis* [33]. Cytochrome P450 (CYP)-mediated detoxification of drugs and xenobiotics requires phase II detoxification enzymes, namely glutathione transferases, for the final degradation process [53]. In addition to these pathways, GSTs are known to play roles in secondary metabolite [54] and auxin metabolism [55]. We mapped the BoGST proteins in the kyoto encyclopedia of genes and genomes (KEGG) database using Blast2Go software [56] to identify their possible roles in the plant. Three major pathways were predicted for BoGST proteins, of which 54 were assigned to glutathione metabolism (map00480), drug metabolism (map00982) and xenobiotics metabolism via cytochrome P450 (map00980). Besides these pathways, BoGST proteins were related to phenylpropanoid biosynthesis (map00940, 18 proteins), ascorbate and aldarate metabolism (map00053, four proteins), arachidonic acid metabolism (map00590, three proteins), tyrosine metabolism (map00350, two proteins), pyruvate metabolism (map00620, four proteins), styrene degradation (map00643, two proteins), and aminoacyl-tRNA biosynthesis (map00970, one protein). Pathway distributions among the different classes of BoGSTs are detailed in Table 3. Overall, the previous pathway-related studies on *GST* genes in plants have reported similar predicted pathways as those for these *BoGST* genes in *B. oleracea*.

Among various GST classes, intron–exon organization is well categorized. The total numbers of exons within the different classes are included Table 2, and individual exons for the genes are listed in Table S3, Figure S2a,b. Members of the largest class, the tau class, contain two exons in their gene structure except *BoGSTU11* (1 exon) (Table S3). The presence of a single exon in this group may possibly be due to selection pressure or genomic duplication. Tau class GSTs were first identified as being encoded by an auxin-inducible gene and are involved in responses to wide range of stresses such as wounding, pathogen infection, herbicides, and extreme temperature. BoGST members of the phi, theta, zeta, TCHQD and mPGES2 classes contained conserved gene orientation with 3, 7, 9, 2, and 6 exons, respectively, in their genomic structure. By contrast, the lambda, DHAR, EF1G, GHR and *GST*2*N* gene structures varied within classes. The results of our predicted intron–exon analysis were similar to those for rice *GST* genes [38]. However, we observed slight deviation when compared to *Arabidopsis* intron–exon GST results [42], which may be due to the genome size and evolution within the *Brassicaceae* family. Zeta GST genes from human, carnation, and *C. elegans* share a common gene structure with three exons, demonstrating that the zeta class gene structure evolved before these lineages diverged, and hence represents the ancestral class [57].

During cold stress, cis-acting factors are involved in activation and overexpression of zeta GST proteins in transgenic rice line [58]. There is no previous report of LTRE (low-temperature-responsive element) regulatory elements in GST superfamily members, although there are other elements such as ABREs (ABA-responsive elements) in wheat *GST* genes [59], an ERE (ethylene-responsive element) in carnation *GST1* [60] and AuxREs (auxin-responsive elements) in tobacco *GST* [61]. To investigate cis-elements in *BoGST* genes, the sequences 1000 bp upstream from the 5′-end of the coding region were analyzed for conserved DRE and LTRE motifs. Nine *BoGST* genes were found to have both putative elements, whereas 10 and 21 genes were predicted to contain either DRE or LTRE cis-acting elements, respectively (Table S6, Figure S3). *BoGSTU1*, *BoGSTU11*, *BoGSTU16*, *BoGSTU19*, *BoGSTL3* and *BoGHR3* contained more putative LTRE elements in the sense strand of the promoter region, whereas only *BoDHAR1*, *BoGST2N-1* and *BomPGES2-1* had LTRE elements in the antisense region of the promoter. Based on computational analysis revealed several regions of DRE and LTRE elements are present in various promoter regions of *BoGST* genes, which might be involved during various abiotic stresses.

Moons et al (2003) reported that *OsGSTU4* and *OsGSTU3* proteins each contain one potential *N*-glycosylation site similar to that of sorghum [62], and that this site is necessary for the activity of GSTs. Prediction using NetNGlyc (Asn-Xaa-Ser/Thr), showed that 33 BoGSTs have a potential site for *N*-glycosylation (Table S3). In addition, protein motif searching identified 10 motifs in BoGST proteins, and motif conservation was higher within the classes than between them (Figure S4a,b). Motif 1, present in 57 BoGSTs, contained the serine active site residue at the 18th position (Figure S5). In rice, out of 79 GSTs, 72 were found to contain a serine residue as their active site in their own motif region [50]. Determination of the sub-cellular localization of a protein is an important step toward learning the function of the protein. Little information regarding GST localization is available in the literature, and due to the lack of targeting peptide sequences in their N-terminal regions [27], most GST proteins are thought to be localized in the cytoplasm. Similarly, we found that most BoGST proteins were predicted to be cytoplasmic by Protcomp (61.5%, 40 proteins), tabulated in Table S3. Understanding the sub-cellular localization of GST proteins will aid in finding other possible proteins associated with GST, as well as determining their associated reactions with other proteins and substrates/ligands.

### 2.3. Chromosomal Distribution, Gene Duplication and Syntenic Regions

**Figure 2:**
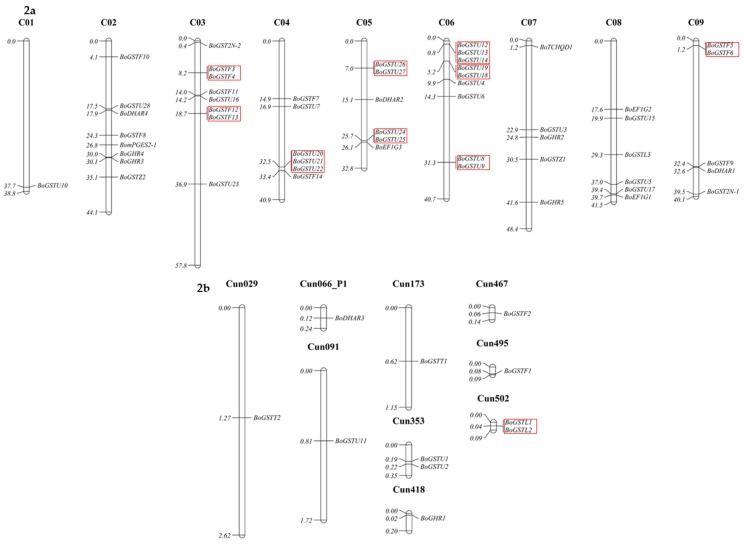
Chromosomal distribution of *GST* genes in *B. oleracea* genome plotted using Mapchart software. The red box indicates genes that are clustered within their GST class.

Analysis of gene distribution in the chromosome showed that *BoGST* genes were spread throughout the genome. The most genes were found in scaffold regions (Cun, 11 genes) and C06 (nine genes), whereas C01 had the fewest *GST* genes (one gene). Among the tau GSTs, 13 were located in C04 and C06, whereas as a total of eight phi GSTs were in C03 and C09 (Figure 2). Clustering of *GST* genes was present only in the largest gene classes tau and phi with six clusters (highest) and three clusters, respectively, whereas we observed one cluster (lowest) in the scaffold regions with lambda GSTs. Similar gene distribution and clustering was reported in *Arabidopsis* [22]. To examine the sequence similarity of BoGST proteins within and between the classes, multiple sequence alignment was performed using DiAlign [63]. As expected, the similarity within the classes was higher than that between classes. GST proteins such as BoGSTU2, BoGSTU6, BoGSTU10, BoGSTU11, BoGSTU23, BoGSTU28, BoGSTF7, BoGSTL3, and BoGHR1 showed less than 60% similarity within their class, compared to not more than 40% between the classes (Tables S7–S10). These results raise the possibility of diversified roles, such as in secondary metabolite metabolism and substrate specificity, within members of a class due to relatively high levels of divergence between the proteins. Thirteen pairs of phi BoGST proteins shared more than 82% similarity, and three pairs of tau BoGSTs had similarity higher than 80%, which indicate high rate of gene duplication within these classes. Such gene duplication might be due to evolutionary pressure imposed on these *GST* gene family or whole genome triplication event in *Brassicaceae* family. As mentioned above, zeta GSTs represent the oldest known class and share high similarity among members from different kingdoms, e.g., humans and carnation share 38% identity, whereas humans and *C. elegans* have 49% similarity. These levels of similarity indicate that significant stretches of sequences have been conserved over a long evolutionary period, thus suggesting common biological function in all organisms [64].

**Figure 3:**
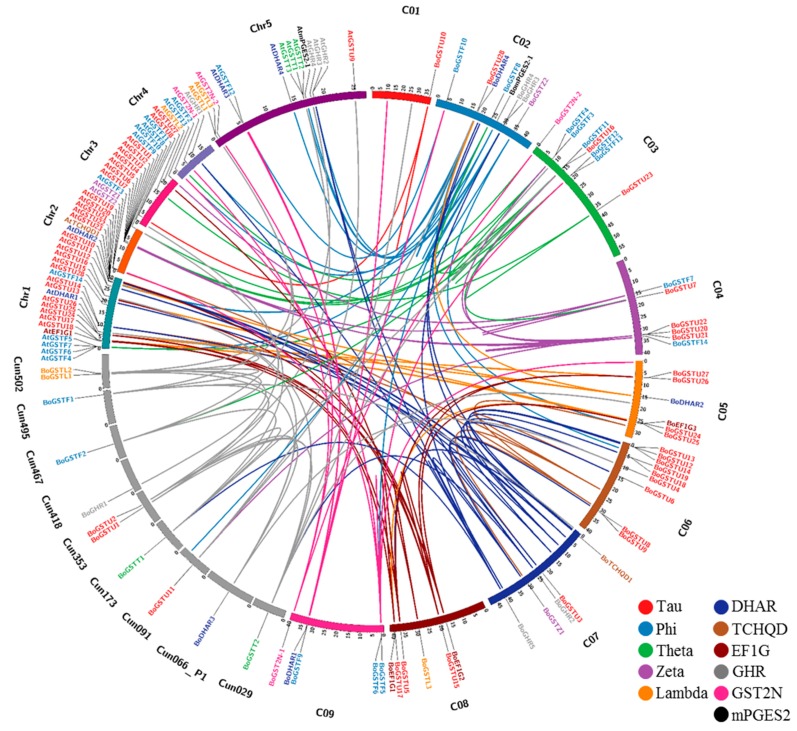
Comparative genome mapping of orthologous and paralogous *GST*’s genes between *B. oleracea* and *Arabidopsis* chromosomes. A high level of conserved syntenic regions between the two species was evident.

The *Brassica* and *Arabidopsis* lineages diverged 20–24 million years ago [65]. To pinpoint the conserved blocks related to GSTs within the *Arabidopsis* and *B. oleracea* genomes, syntenic mapping was examined (Figure 3). Within the *B. oleracea* genome, C06 had more syntenic regions (18.8%) than other chromosomes, whereas C01, with the least (0.1%) *GST* genes showed fewer syntenic regions (Tables S11 and S12). Syntenic regions of *BoGST* genes shared 48.3% similarity (highest) in observed regions with *Arabidopsis* Chr1 (Chromosome 1) and 5.3% (lowest) with Chr4 (chromosome 4). In *Arabidopsis*, Chr1 contains 26 of 55 AtGSTs, which is the reason for high sequence conservation of *BoGST* genes with Chr1, is suggestive of genome evolution of duplicated genes from the same chromosome. Surprisingly, in *Arabidopsis*, Chr5 contains more *GST* genes than Chr4, but there were fewer syntenic regions than in Chr4 [22]. Genome duplication and gene losses during evolution of *Arabidopsis* to *Brassica* are likely responsible for this distribution of synteny between the two species.

### 2.4. Gene-Specific and Organ-Specific Expression Analysis in Non-Treated Samples

**Figure 4:**
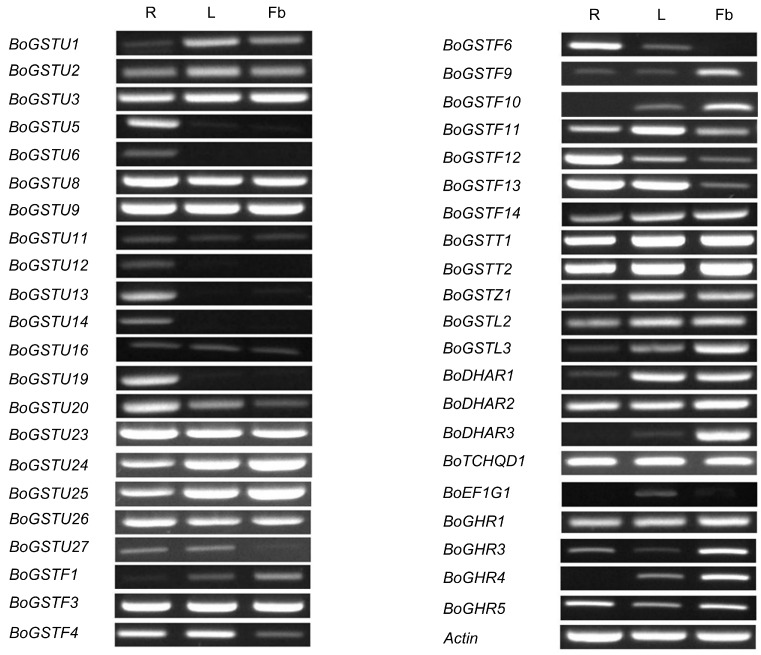
*BoGST* mRNAs showed distinct expression patterns in *B. oleracea* organs (R—Root, L—Leaf, and Fb—Flower bud). Transcripts specific for each *GST* genes amplified by reverse transcription-PCR, visualized using 1.5% agarose gel.

Gene-specific primer pairs for 65 *BoGST*s (Table S13) were used for reverse transcription-PCR (RT-PCR) analysis of class-wise expression patterns of *BoGST*s in different organs (root, leaf, flower buds) of healthy non-treated *B. oleracea* (line *Bo*106). From semi-quantitative RT-PCR analysis among 65 genes, 43 genes were found to express in any one of the investigated tissues (Figure 4). As stated earlier, similarity within the classes was very high, which made it impossible to analyze gene-specific expression of the remaining genes. Of all the expressed genes in organ tissues, 33 genes were expressed in all organs ubiquitously. Notably, *BoGSTU6*, *BoGSTU14* transcripts expressed exclusively in the roots, thus suggesting it is a root-specific gene that could be possibly involved in root associated biological reactions. In rice, tau class *OsGSTU3* and *OsGSTU4* genes are expressed only in root organs and also show high activity against various abiotic stresses [66]. Among the rest, members of all GST classes were expressed in all organs expect EF1G class, *BoGSTF10*, *BoDHAR3*, which were not expressed in root samples. Specifically, *BoGSTF1* was expressed in leaf and flower bud, consistent with its reported function in aliphatic GSL biosynthesis in *B. oleracea* leaves [16]. Overall, the organ-specific expression patterns of *BoGST*s were in parallel with those found in organ-specific microarray expression analysis of *AtGST*s [22] and *OsGST*s [50]. These results shows that *GST* genes are predominantly expressed in all organs suggest that *GST*s may have regulatory functions within developing plant cells.

### 2.5. Differential Expression Pattern under Cold Stress

**Figure 5:**
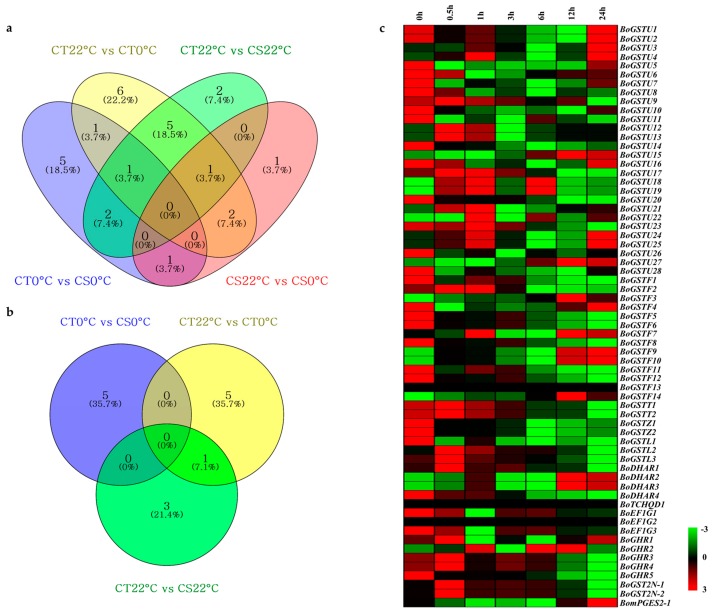
The Venn diagram shows differentially expressed *GST* genes from cold transcriptome data of CT and CS lines: (**a**) up-regulated genes and (**b**) down-regulated genes. Expression profile cluster analysis of *BoGST* superfamily using *Arabidopsis* orthologous microarray data under cold stress: (**c**) aerial (leaf) sample. Expression cluster with red color indicates up-regulated genes and green color indicates down-regulated genes.

*GST* genes are among cold-inducible genes in *Arabidopsis* [47], rice [58], *Solanum* sp. [48], *Euphorbia esula* [32], and *S. bicolor* [67]. Here, cold transcriptome data of the two contrasting lines (cold tolerant (CT) and cold susceptible (CS)) were used for BLAST searches coupled with data mining for our *BoGST* sequences and revealed a total of 33 unigenes differentially expressed during cold stress (Figure 5a,b; Table S14). For further expression profiling analysis based on *Arabidopsis* orthologous genes, cold microarray profiles for aerial parts were downloaded using the AtGenExpress visualization tool to investigate cold stress responses of *GST* genes. A total of 48 orthologous *AtGST* gene expression profiles were obtained for 65 corresponding *BoGST* genes. Relative gene expression using cluster analysis revealed that different class of *GST* genes showed up (red) and down (green) regulation at different time points of cold stress (Figure 5c).

**Figure 6:**
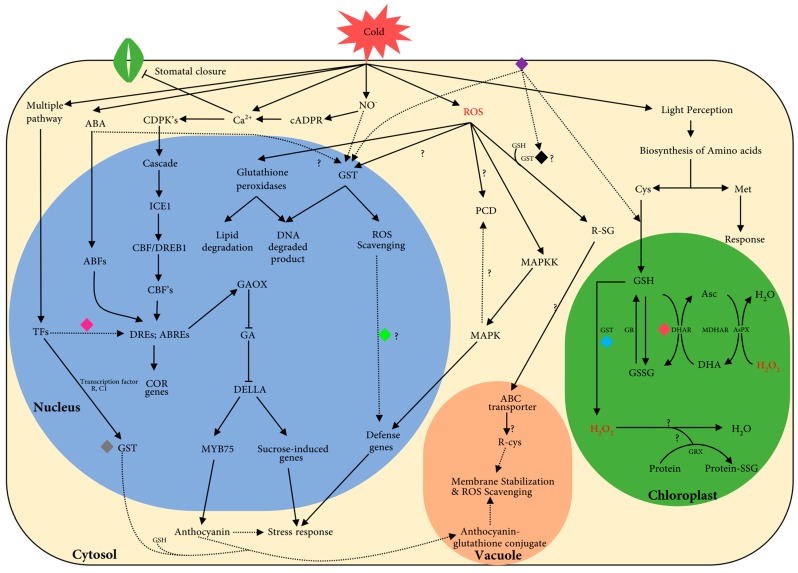
Simplified overview of *GST* genes involved in cold pathway, colored diamond shape represents the possible role of different *GST* classes as mentioned in Table 4. Potential deleterious compound are shown in red color, black solid arrow indicates proven experimental evidence available in literature, black dotted arrow indicates possible predicted function based on localization and protein–protein interaction and the question mark indicates the unknown cellular reaction during cold stress. Role of GSH: photosystem in light perception are proposed pathways for *GST* genes during cold stress. TFs, transcription factor; Transcription factor R bHLH family; C1, R2R3-MYB protein family; ABA, abscisic acid; ABRE, abscisic acid responsive element; COR, cold responsive element; NO^−^, nitric oxide; cADPR, cyclic adenosine triphosphate ribose; Ca^2+^, calcium ion signal; CDPK, calcium dependent protein kinases; ICE1, inducer of CBF expression 1; CBF/DREB1, C-repeat binding factor/dehydration responsive-element binding; DRE, dehydration responsive element; GAOX, gibberellic acid oxidase; GA, gibberellic acid; MYB, myeloblastic transcription factor; ROS, reactive oxygen species; GST, glutathione transferase; PCD, programmed cell death; R-SG, conjugated compound with GSH; MAPK, mitogen-activated protein kinase; MAPKK, MAPK kinase; Cys, cysteine; Met, methionine; GSH, glutathione; GSSG, reduced glutathione; DHAR, dehydroascorbate reductase; DHA, dehydroascorbate; MDHAR- mono dehydroascorbate reductase; Asc, ascorbate; AsPX, ascorbate peroxidase; H_2_O_2_, hydrogen peroxide; GR, glutathione reductase; GRX, glutaredoxin.

Based on our data and previous reports on GST proteins during stress conditions, a simple schematic model for *GST* roles in cold stress is proposed in Figure 6; details for a few genes in different pathways during cold stress are tabulated in Table 4. During cold stress, plant cells sense cold via changes in membrane fluidity and protein conformations, which elicit primary signals, such as ABA, Ca^2+^ (calcium ions), and NO^−^ (nitric oxide) [68], and secondary signals like ROS, stomatal closure, and light perception. The best-known cold response pathway in plants is mediated by transcription factors that bind ABRE and DRE elements in promoters, and further induce *COR* genes (*COR15*, *COR47*, *RD22*, and *RD29A*). In addition, AP2/ERF (apetala2/ethylene response factor) elements are also involved in induction of cold-related genes [5,8,69]. During ROS formation (oxidative burst), GST and GPX proteins are known to be highly induced, and in turn detoxify lipid peroxides, DNA degradation products, and ROS. Further, increased cellular levels of ROS, which are second messengers during cold stress conditions, lead to PCD (programmed cell death) [54]. Besides these functions, ROS are also known to induce MAPKK (mitogen-activated protein kinase kinase) proteins, which further induce defense-related genes via MAPKs, and a conjugation process involving GSH by GST enzyme was further localized in vacuole by transporters (ATP-binding cassette (ABC) transporter cascade) for degradation. In the chloroplast, two types of ROS degradation take place: (i) via the ascorbate-glutathione system mediated by DHAR class members [70]; and (ii) conjugation of GSH aided by GST proteins [71]. ROS degradation in chloroplasts is strongly affected by light perception signal transduction by photosystem, Cys, Met and GSH biosynthesis [70]. Targeting of GST proteins to specific organelles within a plant cell during stress conditions reveals likely roles for those proteins. Based on the expression profile and localization of GST proteins, with especially tau and theta class members targeted inside the nucleus and showing differential gene expression (DGE) in cluster analysis (Figure 5c), some members of the tau and theta class are candidates to play roles as transcription factors [22,55]. By contrast, DHAR protein activity is high in thylakoid membranes of the chloroplast [27] and expression analysis during cold stress showed short-term and long-term induction of genes within the class. In general, cold tolerance of plants positively correlates with levels of anthocyanins, which probably protect chlorophyll from over-excitement during cold stress [13]. However in the phi class, *GSTF12* is regulated by transcription factors R (bHLH family) and C1 (R2R3-MYB protein family), and is involved in translocation of anthocyanins to vacuoles via ABC transporters, which suggests a possible role in membrane stabilization and ROS scavenging during cold stress [72]. Additionally, TCHQD members are plasma membrane proteins whose function is uncharacterized [27], but interact with DHAR, tau, theta and lambda classes in protein–protein interaction network predicted using STRING database (data not shown), suggesting a possible role in signal perception.

**Figure 7:**
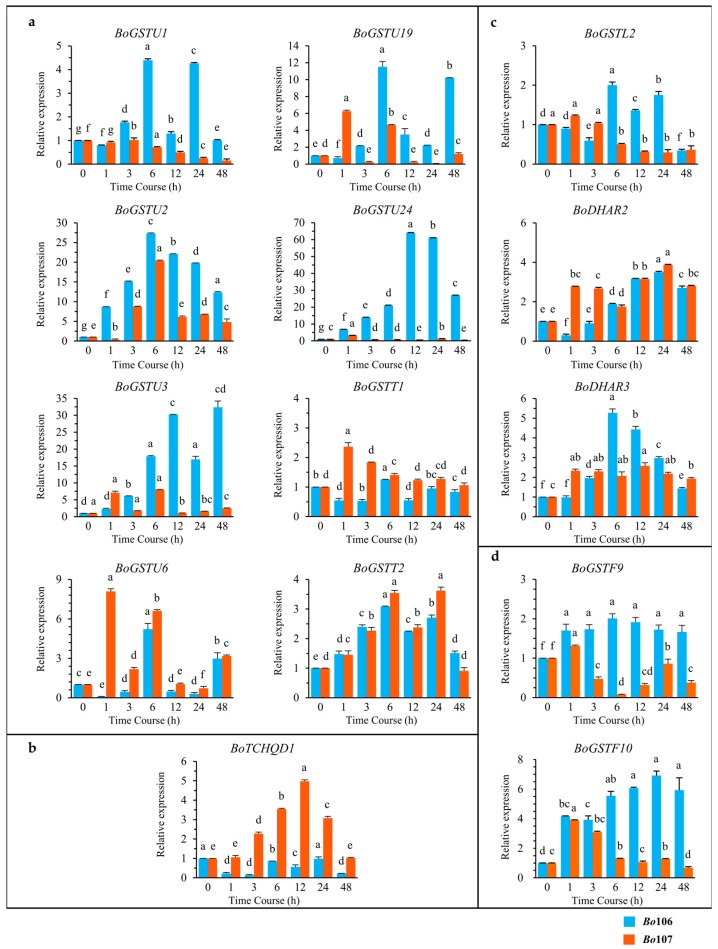
Relative quantitative (RQ) expression analysis of 14 *BoGST* genes which are involved in: (**a**) transcription activation; (**b**) signal perception in plasma membrane; (**c**) H_2_O_2_ reduction in chloroplast; and (**d**) co-regulated genes in anthocyanin sequestration to the vacuoles after cold stress treatment in *Brassica oleracea*. *X*-axis represents Time Course (0, 1, 3, 6, 12, 24, and 48 h) and *Y*-axis represents relative mRNA expression. Graph with orange line is CT line (*Bo*106), blue line is CS line (*Bo*107). For each gene, data are represented as relative expression levels to the levels measured at 0 h (2^−ΔΔ*C*t^). Graph shows the mean of three biological replicates ± standard deviation. (a–e) lowercase letters represent significant differences between different time courses (one-way ANOVA, Tukey’s Test, *p* < 0.05).

Further to validate the expression of *BoGST*s in response to cold, we used leaf samples of two contrasting *B. oleracea* lines, CS and CT, for RT-PCR and qRT-PCR experiments. RT-PCR results for all *GST* genes expressed in the organ were analyzed in cold-treated samples, and all genes were successfully amplified except *BoGSTU14* in leaves of both lines, and *BoGSTU5* in leaf samples of the CS line (Figure S6). Considering previously published results, in silico localization analysis and deduced pathways for *GST* genes during cold stress, we selected *BoGST* genes for further analysis that possibly function as transcription factors (eight genes), H_2_O_2_ reduction in chloroplast (three genes), and signal perception in the plasma membrane (one gene), as well as co-regulated genes in anthocyanin sequestration to the vacuole (two genes). For these genes, qRT-PCR analysis revealed that transcript levels differed in leaf samples along the time course of cold stress (Figure 7). Of the eight genes analyzed based on possible roles at TFs in the GST superfamily (Figure 7a), *BoGSTU2* and *BoGSTT2* showed up-regulation in both inbred lines, showing the highest gene expression at 6 h of cold treatment, followed by down-regulation. Their similar expression patterns in both lines suggest that these two genes are not affected by any varying internal factors. In the CT inbred line, *BoGSTU1*, *BoGSTU3*, *BoGSTU6*, *BoGSTU19* and *BoGSTU24* were positively differentially regulated in response to cold stress; however, high transcript levels of these genes occurred at different times, followed by gradual decreases over the time course. Notably, *BoGSTU3* showed continuous upregulation except at 24 h during cold stress. Moreover, *BoGSTT1* showed no significant expression during stress time. In the CS inbred line, *BoGSTU1*, *BoGSTU19*, *BolGSTU24*, and *BoGSTT1* showed down-regulation along the time period, whereas no significant expression change was observed in *BoGSTU3*, *BoGSTU6* and *BoGSTU19* genes during cold treatment.

For theta class GST proteins, a myb-like transcription factor regulates gene expression during oxidative stress [22]. Similarly, in drought and cold, *GSTT1* in *Euphorbia* *esula* showed higher expression than under control conditions [32]. By contrast, *parA* (tau class) in tobacco possibly functions in transcription regulation in addition to its GST activity [27,55]. In *Arabidopsis*, *AtGSTU7* showed changes in expression within 3 h of different stresses [47]; additionally, *AtGSTU17* acts as negative regulator in drought- and salt-mediated signal transduction [73]. Overall, among putative TFs in the GST superfamily, genes positively expressed were induced early in cold stress, and thus are likely to be positively involved in regulation of cold-related genes, secondary metabolite biosynthesis and metabolism and ROS reduction. Investigation of TCHQD revealed contrasting mRNA transcript levels in CT and CS lines, showing down-regulation and up-regulation respectively, consistent with this gene acting as a negative regulator during cold stress (Figure 7b). However, in rice, *OsTCHQD1* accumulated after 3 h of cold treatment, significantly lower when compared to drought and salt stress [50], and it is also involved in reduction of pesticides [27]. In the *GST* superfamily, few genes encode antioxidant enzymes that reduce ROS produced due to cold stress in chloroplasts. Evaluating transcripts of antioxidant enzymes in *B. oleracea* revealed that *BoDHAR2* was up-regulated in both inbred lines, showing high accumulation at 24 h of cold stress. This activation of *DHAR* genes occurs after high accumulation of ROS levels inside chloroplasts and these genes are also induced after prolonged cold stress (Figure 7c). In the CT line, *BoDHAR3* showed up-regulation with a peak at 6 h, whereas *BoGSTL2* showed no significant expression during the stress period. Different expression patterns were observed for *BoGSTL2* (down-regulation from 3 h) and *BoDHAR3* (no response) in the CS line. In wheat, *DHAR* shows up-regulation in contrasting seasonal varieties during prolonged cold [51], and the level of transcripts was also elevated in transgenic *Arabidopsis* lines compared to wild type under salt stress [74]. In addition, lambda GST activity is increased in response to heavy metals in *Arabidopsis* [30], although there are no reports on other abiotic stresses. Anthocyanin biosynthesis-related genes are induced during cold stress, conferring tolerance to the plants. Anthocyanin biosynthesis-related genes are highly induced during cold stress in kale, a *B. oleracea* member [75], and *GST* genes are also highly induced regulated during high light (HL) stress along with anthocyanin biosynthesis genes in *Arabidopsis* [76]. In *Arabidopsis*, especially *GSTF12* promotes the transport of anthocyanins to vacuoles for increased tolerance during cold stress. Examination of two orthologous genes of *AtGSTF12* in *B. oleracea* revealed that in the CT line, *BoGSTF9* showed static expression and *BoGSTF10* was significantly up-regulated over time during cold stress, whereas the *BoGSTF10* failed to accumulate in the CS line, which might lead to the cold-susceptibility of the plant (Figure 7d). Two-way ANOVA statistical analysis support that expression levels of genes were significant (i.e., significant at 0.1% level of significance) at different time point within and between genotypes (Table S15).

In sum, *BoGSTU24* exhibited strongly contrasting expression patterns in CT and CS line, showing up-regulation and down-regulation, respectively, during cold stress, whereas *BoGSTU19* showed opposite pattern. In CT lines, high accumulation of *GST* transcripts was observed at or after 6 h of cold stress, although in the CS line, the levels were highest at or after 1 h of cold stress. These findings suggest that there are differences in GST gene induction between the two inbred lines, which may be due to induced formation of ROS during cold stress. However, the phenotype and genotype of the two inbred line also affect the transcript levels of *GST* genes as well as ROS levels. In silico analysis of *BoGST* superfamily members supported the transcript expression study of *BoGSTs* in *B. oleracea*. Overall, these findings indicate that *BoGSTU19*, *BolGSTU24*, and *BoGSTF10* are potential genes up-regulated during cold conditions. Further investigation on their functional behavior might help in understanding the cold tolerance mechanism conferred by *GST* superfamily genes. There are also reports on *GST* genes induced through the ABA pathway [29], and the *GST* superfamily may thus also be involved in conferring tolerance to other abiotic stress such as salt, drought, and wounding.

## 3. Materials and Methods

### 3.1. GST Sequence Retrieval

A search was conducted based on annotation for glutathione transferase (*GST*) genes, and corresponding *Bo* (*B. oleracea*) coding (CDS) and protein sequences were retrieved from BRAD [77,78], Bolbase database [79,80] and EnsemblPlants database [81,82]. Furthermore, a complementary method that exploits advanced probabilistic methods, called HMM-profiling, was implemented to increase the accuracy in identifying candidate genes within a genome. For this method, we defined *Arabidopsis* GST amino acid sequences as a primary source of GST-specific domains (GST N- and GST C-domains) in the HMM analysis. Sequences of 55 proteins from *Arabidopsis* with GST-specific domains were aligned using Clustal Omega [83,84]. We used those aligned sequences (Stockholm alignment format) as an input for HMMBUILD program in HMMER 3.1b2 software [85] to construct our GST-specific HMM profile. This user-defined GST-HMM profile was used as model to search against the *B. oleracea* genome acquired from the Bolbase and Ensemble databases using the HMMSEARCH program. Further, the results were subjected to domain analysis using the SMART database [86,87] and CDD (Conserved Domain Database) [88,89] to remove sequences with false domains or partial domain architecture of classic GST proteins. Screening and post-processing of the results were done on the basis of default cutoff values and on the presence of GST-specific domains in their protein structure. The retrieved proteins from the *B. oleracea* genome were revalidated using local BLASTP searches against the NCBI database for confirmation of putative GST functions. The results obtained from GST-specific HMM profile using *Arabidopsis* GSTs were confirmed using another GST-specific profile retrieved from Pfam database (GST_N-PF02798 and GST_C-PF00043).

### 3.2. In Silico Approach for Identification and Characterization of GST Genes

To understand the evolutionary relationship among BoGST proteins, processed GST proteins were aligned using CLUSTALW [90,91] with other known GST sequences from *Arabidopsis*, rice, maize, barley, soybean, wild soybean, and wheat (Table S5) with BLOSUM matrix employing default parameters and the alignment was condensed manually. A molecular phylogenetic tree was constructed using the ML (Maximum likelihood) procedure with the JTT (Jones, Taylor, Thornton) matrix-based amino acid substitution method in MEGA6.06 [92] and 1000 bootstrap replications to access tree topology and reliability. Primary analysis of the predicted molecular weights, pIs, and stability indexes was done using ProtParam [93,94]. Further, *N*-glycosylation sites were predicted using NetNGlyc 1.0 server [95]. Subcellular localization prediction of predicted BoGST proteins was performed using Protcomp 9.0 from Softberry [96]. Secondary structures of GST proteins were predicted using the garnier script tool from EMBOSS-6.6.0 [97]. Motif analysis of proteins was performed using MEME (Multiple Em for Motif Elicitation v4.10.1) [98] with the following parameters: (1) number of motifs = 10; (2) Motif width ≥6 and ≤50. Gene Structure Display Server (GSDS) web tool [99,100] was used to determine the number of introns and exons, using GFF3 (General file format) and aligning CDS and genomic region of the *GST* genes. Prediction of putative cis-acting regulatory elements in *BoGST* genes, using the regions about 1000-bp upstream from the translation initiation site (ATG), was carried out using PlantCARE [101] and PLACE [102], and manually validated as reported by Ibraheem et al. [103].

### 3.3. Chromosomal Location and Syntenic Regions of BoGSTs

*BoGST* gene information, chromosome, gene position, strand, and syntenic regions between *B. oleracea* and *Arabidopsis* were retrieved using the gene locus search option from Bolbase database [80]. Chromosomal positions of *GST* genes were drawn using MapChart 2.3 [104] software program. *GST* genes of *B. oleracea* were aligned against the *B. oleracea* and *Arabidopsis* genome using SyMap v3.4 [105] to obtain syntenic regions within the genome. Subsequently, the derived syntenic regions within the genome and syntenic regions between *B. oleracea GST* genes and the *Arabidopsis* genome from the database were used as input for Circos [106] software for visualization of syntenic regions of *GST* genes.

### 3.4. Sampling and Preparation of Plant Material

To study the expression patterns of *BoGST* genes, two contrasting lines, *Bo*106 (cold tolerant (CT)) and *Bo*107 (cold susceptible (CS)) [8,107] previously referred to as BN106 (cold tolerant (CT)) and BN107 (cold susceptible (CS)) in the reference [5], were grown at the Department of Horticulture, Sunchon National University, Korea. Seeds of the two lines were aseptically inoculated in half-strength murashige and skoog (MS) medium in a growth chamber. The growth chamber was maintained at 25 ± 1 °C for 16 h light/8 h dark conditions. For organ-specific analysis, samples from fresh roots, leaves, and flower buds were removed from three weeks old healthy plants, frozen immediately in liquid nitrogen, and stored at −80 °C until RNA extraction. Three-week-old seedlings were subjected to cold treatment (4 °C) with three replications. Samples of cold-treated leaves were excised at different time points (0, 0.5, 1, 3, 6, 12, 24, and 48 h), frozen immediately in liquid nitrogen, and stored at −80 °C until RNA extraction. Frozen organ samples and cold-treated samples were subjected to total RNA extraction using an RNeasy Mini Kit (Qiagen, Valencia, CA, USA), subsequently RNA cleanup by DNase I treatment, (Takara Bio, Inc., Shiga, Japan). Isolated RNA was quantified using an ND-1000 Spectrophotometer and NanoDrop v3.7 software (NanoDrop Technologies, Wilmington, DE, USA). Synthesis of cDNA from RNA extracts was performed with Superscript III^®^ First-strand Synthesis Supermix kit (Invitrogen, Carlsbad, CA, USA) following the manufacturer’s instructions.

### 3.5. Qualitative and Quantitative PCR Expression Analysis

Initial analysis of expression patterns was carried out using microarray data of *Arabidopsis* with orthologous loci downloaded from the AtGenExpress visualization tool (AVT) [108] during cold stress (leaf samples). Expression cluster analysis of *GST* genes were performed with the Cluster program [109] and results were visualized using GenePattern software [56,110]. Additionally, cold transcriptome data of *B. oleracea* for *Bo*106 (CT) and *Bo*107 (CS) lines were downloaded from the NCBI database [111] using TSA (Transcriptome Shotgun assembly; GAQY00000000) and SRA (Sequence Read Archive; SRS490050). Further, qualitative expression analysis using RT-PCR was conducted using one-step EmeraldAmp GT PCR Master Mix (Takara, Bio, Inc., Shiga, Japan). Gene-specific primer pairs for *BoGST*s were used for RT-PCR and the actin gene from *B. oleracea* (JQ435879) was used as a housekeeping control gene (Table S13). RT-PCR was performed using 50 ng cDNA (1 µL) from organ and cold-treated samples as template in a master mix consisting of 2 µL primer pairs (10 pmol each of forward and reverse primer), 8 µL sterile water, and 9 µL Emerald master mix, in a total volume of 20 µL. PCR conditions were set as follows: initial denaturation 94 °C, succeeded by 35 cycles (30 cycles for organ samples) of denaturation at 94 °C for 30 s, annealing at 60 °C for 30 s, and extension at 72 °C for 45 s, with a final extension of 5 min at 72 °C for cold-treated samples. PCR products were visualized using 1.5% agarose gels (Duchefa Biochemie, Haarlem, The Netherlands).

Real-Time quantitative PCR (qRT-PCR) was executed using 1 µL cDNA in a 20-µL reaction volume with iTaqTM SYBR^®^ Green Super-mix with ROX (Foster City, CA, USA). Class-wise gene-specific primers for qRT-PCR were employed in this experiment (Table S13). Thermal-cycler conditions were set as follows: 5 min at 95 °C, followed by 40 cycles at 95 °C for 10 s, 60 °C for 10 s, 72 °C for 20 s, and then melting at 72 °C for 60 s and 97 °C for 1 s. The fluorescence was assessed following the last step of each cycle. Product amplification, detection, and data inspection were carried out using LightCycler96 (Roche, Basel, Switzerland). Relative gene expression levels were calculated using the ∆∆*C*_t_ method. Actin was used as housekeeping gene.

### 3.6. Data Statistics

Statistical data analysis was performed for the relative gene expression levels from three biological replicates under each treatment (time-point) × genotype (inbred line) combinations. The log-transformed values were analyzed by two-way analysis of variance (ANOVA) following a generalized linear model using the MINITAB 16 (Minitab Inc., State College, PA, USA) statistical software. To separate the means under each treatment, a Tukey’s pairwise comparison test was performed.

## 4. Conclusions

This is the first report on genome-wide characterization of *GST*s in *B. oleracea*. In short, using a combined computational strategy, we identified 65 *BoGST*s in the *B. oleracea* genome and characterized them based on domain, gene, and protein structures, sequence similarities, and expression patterns in response to cold stress conditions. Using two contrasting lines, *BoGST* genes were found to possess potential functions against cold stress in *B. oleracea*. Overall, the roles of *GST* along with GSH conjugation in various pathways and degradation process are important to consider for engineering of these candidates gene in recombinant DNA technology for the development of suitable and elite transgenic cultivars that can withstand various abiotic stresses.

## Figures and Tables

**Table 1:** Number of GST genes content in *Brassica oleracea*, *Arabidopsis thaliana*, *Hordeum vulgare*, *Populus trichocarpa*, *Solanum lycopersicum*, *Oryza sativa*, *Zea mays* and *Glycine max*.

Plant	*Brassica oleracea*	*Arabidopsis thaliana*	*Hordeum vulgare*	*Populus trichocarpa*	*Solanum lycopersicum*	*Oryza sativa*	*Zea mays*	*Glycine max*
GST Family	Number	Number	Number	Number	Number	Number	Number	Number
Tau	28	28	50	58	56	40	28	20
Phi	14	13	21	9	5	16	12	4
Theta	2	2	1	2	4	2	N/A ^a^	N/A ^a^
Zeta	2	2	5	2	2	3	2	1
Lambda	3	3	2	3	5	N/A ^a^	N/A ^a^	N/A ^a^
DHAR	4	4	2	3	6	N/A ^a^	N/A ^a^	N/A ^a^
TCHQD	1	1	1	1	1	N/A ^a^	N/A ^a^	N/A ^a^
EF1G	3	2	2	3	1	N/A ^a^	N/A ^a^	N/A ^a^
Others	8	N/A ^a^	N/A ^a^	N/A ^a^	1	N/A ^a^	N/A ^a^	N/A ^a^
Total	65	55	84	81	81	61	42	25
Reference		[44]	[36]	[40]	[39]	[38]	[37]	[37]

^a^, not available; GST, glutathione transferase; DHAR, dehydroascorbate reductase; TCHQD, tetrachlorohydroquinone dehalogenase; EF1G, elongation factor 1 γ.

**Table 2:** Characterization of *GST* genes in *Brassica oleracea*.

Sr. No.	Class	No. of *GST* Genes	Nucleotide Length Range (bp)	ORF Range (bp)	No. of Exons	Protein					
						Length Range (aa)	Mol. Wt. Range (kDa)	pI Range	Average Domain Range		
									GST N-Region	GST C-Region	EF1G Region
1	Tau	28	705–4093	570–942	1–2	189–313	21.56–35.13	4.96–8.85	72–75	110–146	-
2	Phi	14	780–1198	603–777	3	200–258	22.59–28.84	5.13–8.21	60–75	114–118	-
3	Theta	2	1406–1470	726–738	7	241–245	27.38–27.66	9.36–9.5	75	128	-
4	Zeta	2	1994–2097	591–714	9	196–237	22.26–26.35	5.29–6.91	44–77	118–119	-
5	Lambda	3	1430–1776	708–906	8–9	187–301	21.37–34.39	5.08–8.82	77–68	88–121	-
6	DHAR	4	851–1421	633–774	3–6	210–257	23.22–28.63	5.76–8.28	56–72	118–121	-
7	TCHQD	1	1071	801	2	266	31.46	9.26	72	99	-
8	EF1G	3	1902–2326	1239–1248	6–7	412–415	46.4–46.57	5.56–5.64	71–81	107–120	106–108
9	GHR	5	1290–1576	954–1212	3–5	317–403	36.43–44.9	6.32–8.2	88–106	111–141	-
10	GST2N	2	2196–2401	1011–1017	11–12	336–338	36.93–36.95	8.81–9.26	76–77	-	-
11	mPGES2	1	1464	942	6	313	35.13	8..85	72	146	-

Sr. No., serial number; bp, base pair; Mol. Wt., molecular weight; aa, amino acid; kDa, Kilodalton; pI, Iso-electric point; GST, Glutathione transferase; N-, N-terminal; C-, C-terminal; EF1G, Elongation factor 1 γ.

**Table 3:** Organization of *GST* super-family in *B. oleracea* based on pathway analysis using KEGG database.

Class	Abbreviation	Genes	Predicted Function
Tau	GSTU	28	Drug and Xenobiotics metabolism-cytochrome P450, Glutathione metabolism, Pyruvate metabolism, Phenylpropanoid biosynthesis
Phi	GSTF	14	Drug and Xenobiotics metabolism-cytochrome P450, Glutathione metabolism, Pyruvate metabolism, Phenylpropanoid biosynthesis, Arachidonic acid metabolism
Theta	GSTT	2	Drug and Xenobiotics metabolism-cytochrome P450, Glutathione metabolism, Phenylpropanoid biosynthesis
Zeta	GSTZ	2	Drug metabolism-cytochrome P450, Glutathione metabolism, Styrene degradation, Tyrosine and Pyruvate metabolism
Lambda	GSTL	3	Drug and Xenobiotics metabolism-cytochrome P450, Glutathione metabolism
DHAR	DHAR	4	Drug and Xenobiotics metabolism-cytochrome P450, Glutathione metabolism, Pyruvate metabolism, Phenylpropanoid biosynthesis, Ascorbate and aldarate metabolism, Aminoacyl-tRNA biosynthesis
TCHQD	TCHQD	1	Drug and Xenobiotics metabolism-cytochrome P450, Glutathione metabolism
EF1G	EF1G	3	N/A ^a^
GHR	GHR	5	Drug and Xenobiotics metabolism-cytochrome P450, Glutathione metabolism
GST2N	GST2N	2	N/A ^a^
mPGES2	mPGES2	1	N/A ^a^

^a^, Not available.

**Table 4:** Predicted *BoGST* genes involving in various pathways during cold stress in *B. oleracea*.

Sr. No.	Image	Possible Role	Localization	GST Family	Possible GST Class Involvement
1	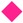	TFs	Nucleus	Tau and Theta	*BoGSTU1*, *BoGSTU2*, *BoGSTU3*, *BoGSTU6*, *BoGSTU10*, *BoGSTU17*, *BoGSTU18*, *BoGSTU19*, *BoGSTU24*, *BoGSTU25*, *BoGSTT1*, *BoGSTT2*
2	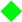	Gene induction	Nucleus	Unknown	Unknown classes
3	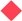	H_2_O_2_ Reduction	Chloroplast	DHAR	*BoDHAR1*, *BoDHAR2*, *BoDHAR3*, *BoDHAR4*
4	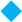	H_2_O_2_ Reduction	Chloroplast	Lambda	*BoGSTL1*, *BoGSTL2*
5	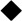	GSH conjugation	Cytoplasm	Unknown	Unknown classes
6	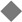	Anthocyanin-transporting gene	Cytoplasm	Phi	*BoGSTF9*, *BoGSTF10*
7	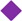	Signaling protein	Plasma membrane	TCHQD	*BoTCHQD1*

## Supplementary Materials

Supplementary materials can be found at http://www.mdpi.com/1422-0067/17/8/1211/s1.

## Abbreviations

ABA	Abscisic acid
BoGST	*Brassica oleracea* Glutathione transferases
CS	Cold susceptible
CT	Cold tolerant
DHAR	Dehydro ascorbate reductase
GHR	Glutathionyl Hydroquinone reductase
GPX	Glutathione peroxidases
GR	Glutathione reductase
GSH	Glutathione
GST	Glutathione transferases
GST2N	GST protein with two repeats of thioredoxin domain
mPGES2	Microsomal Prostaglandin E Synthase type 2
ROS	Reactive oxygen species
SOD	Superoxide dismutase

## References

[1] Thomashow M.F. (1999). Plant cold acclimation: Freezing tolerance genes and regulatory mechanisms. Annu. Rev. Plant Physiol. Plant Mol. Biol..

[2] Yamaguchi-Shinozaki K., Shinozaki K. (2006). Transcriptional regulatory networks in cellular responses and tolerance to dehydration and cold stresses. Annu. Rev. Plant Biol..

[3] Yamaguchi-Shinozaki K., Shinozaki K. (2005). Organization of cis-acting regulatory elements in osmotic- and cold-stress-responsive promoters. Trends Plant Sci..

[4] Lee B., Lee H., Xiong L., Zhu J.-K. (2002). A mitochondrial complex I defect impairs cold-regulated nuclear gene expression. Plant Cell.

[5] Ahmed N.U., Jung H.-J., Park J.-I., Cho Y.-G., Hur Y., Nou I.-S. (2015). Identification and expression analysis of cold and freezing stress responsive genes of Brassica oleracea. Gene.

[6] Du C., Hu K., Xian S., Liu C., Fan J., Tu J., Fu T. (2016). Dynamic transcriptome analysis reveals AP2/ERF transcription factors responsible for cold stress in rapeseed (*Brassica napus* L.) factors responsible for cold stress in rapeseed (*Brassica napus* L.). Mol. Genet. Genom..

[7] Hwang I., Jung H.-J., Park J.-I., Yang T.-J., Nou I.-S. (2014). Transcriptome analysis of newly classified bZIP transcription factors of *Brassica rapa* in cold stress response. Genomics.

[8] Thamilarasan S.K., Park J.-I., Jung H.-J., Nou I.-S. (2014). Genome-wide analysis of the distribution of AP2/ERF transcription factors reveals duplication and CBFs genes elucidate their potential function in *Brassica oleracea*. BMC Genom..

[9] Livingston D.P., Premakumar R., Tallury S.P. (2006). Carbohydrate partitioning between upper and lower regions of the crown in oat and rye during cold acclimation and freezing. Cryobiology.

[10] Kovács Z., Simon-Sarkadi L., Szucs A., Kocsy G. (2010). Differential effects of cold, osmotic stress and abscisic acid on polyamine accumulation in wheat. Amino Acids.

[11] Christie P.J., Alfenito M.R., Walbot V. (1994). Impact of low-temperature stress on general phenylpropanoid and anthocyanin pathways: Enhancement of transcript abundance and anthocyanin pigmentation in maize seedlings. Planta.

[12] Baskar V., Gururani M.A., Yu J.W., Park S.W. (2012). Engineering glucosinolates in plants: Current knowledge and potential uses. Appl. Biochem. Biotechnol..

[13] Janska A., Maršík P., Zelenková S., Ovesná J. (2010). Cold stress and acclimation-what is important for metabolic adjustment?. Plant Biol..

[14] Chinnusamy V., Zhu J., Zhu J.-K. (2007). Cold stress regulation of gene expression in plants. Trends Plant Sci..

[15] Miura K., Jin J.B., Lee J., Yoo C.Y., Stirm V., Miura T., Ashworth E.N., Bressan R.A., Yun D.-J., Hasegawa P.M. (2007). SIZ1-mediated sumoylation of ICE1 controls CBF3/DREB1A expression and freezing tolerance in Arabidopsis. Plant Cell.

[16] Liu S., Liu Y., Yang X., Tong C., Edwards D., Parkin I.A.P., Zhao M., Ma J., Yu J., Huang S. (2014). The Brassica oleracea genome reveals the asymmetrical evolution of polyploid genomes. Nat. Commun..

[17] Kitamura S., Shikazono N., Tanaka A. (2004). TRANSPARENT TESTA 19 is involved in the accumulation of both anthocyanins and proanthocyanidins in Arabidopsis. Plant J..

[18] Alfenito M.R., Souer E., Goodman C.D., Buell R., Mol J., Koes R., Walbot V. (1998). Functional complementation of anthocyanin sequestration in the vacuole by widely divergent glutathione *S*-transferases. Plant Cell.

[19] Dixon D.P., Cummins I., Cole D.J., Edwards R. (1998). Glutathione-mediated detoxification systems in plants. Curr. Opin. Plant Biol..

[20] Oakley A. (2011). Glutathione transferases: A structural perspective. Drug Metab. Rev..

[21] Lallement P.-A., Brouwer B., Keech O., Hecker A., Rouhier N. (2014). The still mysterious roles of cysteine-containing glutathione transferases in plants. Front. Pharmacol..

[22] Dixon D.P., Edwards R. (2010). Glutathione transferases. Arabidopsis Book.

[23] Edwards R., Dixon D.P. (2005). Plant glutathione transferases. Methods Enzymol..

[24] Agrawal G.K., Jwa N.-S., Rakwal R. (2002). A pathogen-induced novel rice (*Oryza sativa* L.) gene encodes a putative protein homologous to type II glutathione *S*-transferases. Plant Sci..

[25] Mueller L.A., Goodman C.D., Silady R.A., Walbot V. (2000). AN9, a petunia glutathione *S*-transferase required for anthocyanin sequestration, is a flavonoid-binding protein. Plant Physiol..

[26] Kampranis S.C., Damianova R., Atallah M., Toby G., Kondi G., Tsichlis P.N., Makris A.M. (2000). A novel plant glutathione *S*-transferase/peroxidase suppresses Bax lethality in yeast. J. Biol. Chem..

[27] Dixon D.P., Hawkins T., Hussey P.J., Edwards R. (2009). Enzyme activities and subcellular localization of members of the arabidopsis glutathione transferase superfamily. J. Exp. Bot..

[28] Dixon D.P., Sellars J.D., Edwards R. (2011). The *Arabidopsis* phi class glutathione transferase AtGSTF2: Binding and regulation by biologically active heterocyclic ligands. Biochem. J..

[29] Moons A. (2005). Regulatory and functional interactions of plant growth regulators and plant glutathione *S*-transferases (GSTs). Vitam. Horm..

[30] Dixon D.P., Davis B.G., Edwards R. (2002). Functional divergence in the glutathione transferase superfamily in plants: Identification of two classes with putative functions in redox homeostasis in *Arabidopsis thaliana*. J. Biol. Chem..

[31] Dixon D.P., Lapthorn A., Edwards R. (2002). Plant glutathione transferases. Genome Biol..

[32] Anderson J.V., Davis D.G. (2004). Abiotic stress alters transcript profiles and activity of glutathione *S*-transferase, glutathione peroxidase, and glutathione reductase in Euphorbia esula. Physiol. Plant..

[33] Dixon D.P., Cole D.J., Edwards R. (2000). Characterisation of a zeta class glutathione transferase from *Arabidopsis thaliana* with a putative role in tyrosine catabolism. Arch. Biochem. Biophys..

[34] Keck A.-S., Finley J.W. (2004). Cruciferous vegetables: Cancer protective mechanisms of glucosinolate hydrolysis products and selenium. Integr. Cancer Ther..

[35] Armstrong R.N. (1997). Structure, catalytic mechanism, and evolution of the glutathione transferases. Chem. Res. Toxicol..

[36] Rezaei M.K., Shobbar Z.-S., Shahbazi M., Abedini R., Zare S. (2013). Glutathione *S*-transferase (GST) family in barley: Identification of members, enzyme activity, and gene expression pattern. J. Plant Physiol..

[37] McGonigle B., Keeler S.J., Lau S.C., Koeppe M.K., Keefe D.P.O. (2000). A Genomics Approach to the comprehensive analysis of the glutathione *S*-transferase gene family in soybean and maize. Plant Physiol..

[38] Soranzo N., Sari Gorla M., Mizzi L., de Toma G., Frova C. (2004). Organisation and structural evolution of the rice glutathione *S*-transferase gene family. Mol. Genet. Genom..

[39] Csiszar J., Horvath E., Vary Z., Galle A., Bela K., Brunner S., Tari I. (2014). Glutathione transferase supergene family in tomato: Salt stress-regulated expression of representative genes from distinct GST classes in plants primed with salicylic acid. Plant Physiol. Biochem..

[40] Lan T., Yang Z.-L., Yang X., Liu Y.-J., Wang X.-R., Zeng Q.-Y. (2009). Extensive functional diversification of the populus glutathione *S*-transferase supergene family. Plant Cell.

[41] Sheehan D., Meade G., Foley V.M., Dowd C.A. (2001). Structure, function and evolution of glutathione transferases: Implications for classification of non-mammalian members of an ancient enzyme superfamily. J. Biochem..

[42] Frova C. (2003). The plant glutathione transferase gene family: Genomic structure, functions, expression and evolution. Physiol. Plant..

[43] Mohsenzadeh S., Esmaeili M., Moosavi F., Shahrtash M. (2011). Plant glutathione *S*-transferase classification, structure and evolution. Afr. J. Biotechnol..

[44] Wagner U., Edwards R., Dixon D.P., Mauch F. (2002). Probing the diversity of the *Arabidopsis* glutathione *S*-transferase gene family. Plant Mol. Biol..

[45] Droog F. (1997). Plant glutathione *S*-transferases, a tale of theta and tau. J. Plant Growth Regul..

[46] Edwards R., Dixon D.P., Walbot V. (2000). Plant glutathione *S*-transferases: Enzymes with multiple functions in sickness and in health. Trends Plant Sci..

[47] Sappl P.G., Carroll A.J., Clifton R., Lister R., Whelan J., Harvey Millar A., Singh K.B. (2009). The *Arabidopsis* glutathione transferase gene family displays complex stress regulation and co-silencing multiple genes results in altered metabolic sensitivity to oxidative stress. Plant J..

[48] Seppänen M.M., Cardi T., Borg Hyökki M., Pehu E. (2000). Characterization and expression of cold-induced glutathione *S*-transferase in freezing tolerant *Solanum commersonii*, sensitive *S. tuberosum* and their interspecific somatic hybrids. Plant Sci..

[49] Tsuchiya T., Takesawa T., Kanzaki H., Nakamura I. (2004). Genomic structure and differential expression of two tandem-arranged GSTZ genes in rice. Gene.

[50] Jain M., Ghanashyam C., Bhattacharjee A. (2010). Comprehensive expression analysis suggests overlapping and specific roles of rice glutathione *S*-transferase genes during development and stress responses. BMC Genom..

[51] Baek K.-H., Skinner D.Z. (2003). Alteration of antioxidant enzyme gene expression during cold acclimation of near-isogenic wheat lines. Plant Sci..

[52] Ahsan N., Lee D.G., Alam I., Kim P.J., Lee J.J., Ahn Y.O., Kwak S.S., Lee I.J., Bahk J.D., Kang K.Y. (2008). Comparative proteomic study of arsenic-induced differentially expressed proteins in rice roots reveals glutathione plays a central role during As stress. Proteomics.

[53] Kumar S., Jin M., Weemhoff J. (2012). Cytochrome P450-mediated phytoremediation using transgenic plants: A need for engineered cytochrome P450 enzymes. J. Pet. Environ. Eng..

[54] Marrs K.A. (1996). The functions and regulation of glutathione *S*-transferases in plants. Annu. Rev. Plant Physiol. Plant Mol. Biol..

[55] Takahashi Y., Hasezawa S., Kusaba M., Nagata T. (1995). Expression of the auxin-regulated *parA* gene in transgenic tobacco and nuclear localization of its gene products. Planta.

[56] Blast2GO. https://www.blast2go.com/.

[57] Blackburn A.C., Woollatt E., Sutherland G.R., Board P.G. (1998). Characterization and chromosome location of the gene GSTZ1 encoding the human Zeta class glutathione transferase and maleylacetoacetate isomerase. Cytogenet. Cell Genet..

[58] Takesawa T., Ito M., Kanzaki H., Kameya N., Nakamura I. (2002). Over-expression of ζ glutathione *S*-transferase in transgenic rice enhances germination and growth at low temperature. Mol. Breed..

[59] Pandey B., Sharma P., Pandey D.M., Varshney J., Sheoran S., Singh M., Singh R., Sharma I., Chatrath R. (2012). Comprehensive computational analysis of different classes of glutathione *S*-transferases in *Triticum aestivum* L.. Plant Omics J..

[60] Itzhaki H., Maxson J.M., Woodson W.R. (1994). An ethylene-responsive enhancer element is involved in the senescence-related expression of the carnation glutathione-*S*-transferase (GST1) gene. Proc. Natl. Acad. Sci. USA.

[61] Van der Zaal B.J., Droog F.N., Pieterse F.J., Hooykaas P.J. (1996). Auxin-sensitive elements from promoters of tobacco GST genes and a consensus as-1-like element differ only in relative strength. Plant Physiol..

[62] Gronwald J.W., Plaisance K.L. (1998). Isolation and characterization of glutathione *S*-transferase isozymes from sorghum. Plant Physiol..

[63] Genomatix Software Suit. http://www.genomatix.de/cgi-bin/dialign/dialign.pl.

[64] Board P.G., Baker R.T., Chelvanayagam G., Jermiin L.S. (1997). Zeta, a novel class of glutathione transferases in a range of species from plants to humans. Biochem. J..

[65] Ziolkowski P.A., Kaczmarek M., Babula D., Sadowski J. (2006). Genome evolution in *Arabidopsis/Brassica:* Conservation and divergence of ancient rearranged segments and their breakpoints. Plant J..

[66] Moons A. (2003). Osgstu3 and osgtu4, encoding tau class glutathione *S*-transferases, are heavy metal- and hypoxic stress-induced and differentially salt stress-responsive in rice roots. FEBS Lett..

[67] Chi Y., Cheng Y., Vanitha J., Kumar N., Ramamoorthy R., Ramachandran S., Jiang S.-Y. (2011). Expansion mechanisms and functional divergence of the glutathione *S*-transferase family in sorghum and other higher plants. DNA Res..

[68] Chinnusamy V., Zhu J., Zhu J.K. (2006). Gene regulation during cold acclimation in plants. Physiol. Plant..

[69] Jung H.-J., Dong X., Park J.-I., Thamilarasan S.K., Lee S.S., Kim Y.-K., Lim Y.-P., Nou I.-S., Hur Y. (2014). Genome-wide transcriptome analysis of two contrasting brassica rapa doubled haploid lines under cold-stresses using Br135K oligomeric chip. PLoS ONE.

[70] Tausz M., Šircelj H., Grill D. (2004). The glutathione system as a stress marker in plant ecophysiology: Is a stress-response concept valid?. J. Exp. Bot..

[71] Zagorchev L., Seal C.E., Kranner I., Odjakova M. (2013). A central role for thiols in plant tolerance to abiotic stress. Int. J. Mol. Sci..

[72] Petrussa E., Braidot E., Zancani M., Peresson C., Bertolini A., Patui S., Vianello A. (2013). Plant flavonoids-biosynthesis, transport and involvement in stress responses. Int. J. Mol. Sci..

[73] Chen J.-H., Jiang H.-W., Hsieh E.-J., Chen H.-Y., Chien C.-T., Hsieh H.-L., Lin T.-P. (2012). drought and salt stress tolerance of an *Arabidopsis* glutathione *S*-transferase U17 knockout mutant are attributed to the combined effect of glutathione and abscisic acid. Plant Physiol..

[74] Ushimaru T., Nakagawa T., Fujioka Y., Daicho K., Naito M., Yamauchi Y., Nonaka H., Amako K., Yamawaki K., Murata N. (2006). Transgenic *Arabidopsis* plants expressing the rice dehydroascorbate reductase gene are resistant to salt stress. J. Plant Physiol..

[75] Zhang B., Hu Z., Zhang Y., Li Y., Zhou S., Chen G. (2012). A putative functional MYB transcription factor induced by low temperature regulates anthocyanin biosynthesis in purple kale (*Brassica Oleracea* var. *acephala f. tricolor*). Plant Cell Rep..

[76] Vanderauwera S., Zimmermann P., Van Breusegem F., Langebartels C., Gruissem W., Inze D., Breusegem F. (2005). Van Genome-wide analysis of hydrogen peroxide-regulated gene expression in *Arabidopsis* reveals a high light-induced transcriptional cluster involved in anthocyanin biosynthesis. Plant Physiol..

[77] Cheng F., Liu S., Wu J., Fang L., Sun S., Liu B., Li P., Hua W., Wang X. (2011). BRAD, the genetics and genomics database for *Brassica* plants. BMC Plant Biol..

[78] *Brassica* Database. http://brassicadb.org/brad/.

[79] Yu J., Zhao M., Wang X., Tong C., Huang S., Tehrim S., Liu Y., Hua W., Liu S. (2013). Bolbase: A comprehensive genomics database for *Brassica oleracea*. BMC Genom..

[80] Bolbase Database. http://www.ocri-genomics.org/bolbase/.

[81] Kersey P.J., Allen J.E., Christensen M., Davis P., Falin L.J., Grabmueller C., Hughes D.S.T., Humphrey J., Kerhornou A., Khobova J. (2011). Fast, scalable generation of high-quality protein multiple sequence alignments using Clustal Omega. Mol. Syst. Biol..

[82] EnsemblPlants Database. http://plants.ensembl.org/Brassica_oleracea/Info/Index.

[83] Eddy S.R. (2009). A new generation of homology search tools based on probabilistic inference. Genome Inform..

[84] Clustal Omega. http://www.ebi.ac.uk/Tools/msa/clustalo/.

[85] Schultz J., Milpetz F., Bork P., Ponting C.P. (1998). SMART, a simple modular architecture research tool: Identification of signaling domains. Proc. Natl. Acad. Sci. USA.

[86] Marchler-Bauer A., Derbyshire M.K., Gonzales N.R., Lu S., Chitsaz F., Geer L.Y., Geer R.C., He J., Gwadz M., Hurwitz D.I. (2015). CDD: NCBI’s conserved domain database. Nucleic Acids Res..

[87] SMART Database. http://smart.embl-heidelberg.de/.

[88] Thompson J.D., Higgins D.G., Gibson T.J. (1994). CLUSTAL W: Improving the sensitivity of progressive multiple sequence alignment through sequence weighting, position-specific gap penalties and weight matrix choice. Nucleic Acids Res..

[89] Conserved Domain Database. http://www.ncbi.nlm.nih.gov/Structure/cdd/cdd.shtml.

[90] Tamura K., Stecher G., Peterson D., Filipski A., Kumar S. (2013). MEGA6: Molecular evolutionary genetics analysis version 6.0. Mol. Biol. Evol..

[91] CLUSTALW. http://www.genome.jp/tools/clustalw/.

[92] Gasteiger E., Hoogland C., Gattiker A., Duvaud S., Wilkins M.R., Appel R.D., Bairoch A. (2005). Protein identification and analysis tools on the ExPASy server. The Proteomics Protocols Handbook.

[93] Gupta R., Jung E., Brunak S. (2004). Prediction of *N*-glycosylation sites in human proteins.

[94] ProtParam. http://web.expasy.org/protparam/.

[95] Bailey T.L., Williams N., Misleh C., Li W.W. (2006). MEME: Discovering and analyzing DNA and protein sequence motifs. Nucleic Acids Res..

[96] Softberry. http://linux1.softberry.com/berry.phtml.

[97] EMBOSS-6.6.0. http://emboss.sourceforge.net/apps/.

[98] Hu B., Jin J., Guo A.-Y., Zhang H., Luo J., Gao G. (2014). GSDS 2.0: An upgraded gene feature visualization server. Bioinformatics.

[99] Rombauts S., Déhais P., Van Montagu M., Rouzé P. (1999). PlantCARE, a plant cis-acting regulatory element database. Nucleic Acids Res..

[100] Gene Structure Display Server (GSDS) Web Tool. http://gsds.cbi.pku.edu.cn/index.php.

[101] Higo K., Ugawa Y., Iwamoto M., Korenaga T. (1999). Plant cis-acting regulatory DNA elements (PLACE) database: 1999. Nucleic Acids Res..

[102] Ibraheem O., Botha C.E.J., Bradley G. (2010). In silico analysis of cis-acting regulatory elements in 5′ regulatory regions of sucrose transporter gene families in rice (Oryza sativa Japonica) and *Arabidopsis thaliana*. Comput. Biol. Chem..

[103] Voorrips R.E. (2002). MapChart: Software for the graphical presentation of linkage maps and QTLs. J. Hered..

[104] Soderlund C., Bomhoff M., Nelson W.M. (2011). SyMAP v3.4: A turnkey synteny system with application to plant genomes. Nucleic Acids Res..

[105] Krzywinski M., Schein J., Birol I., Connors J., Gascoyne R., Horsman D., Jones S.J., Marra M.A. (2009). Circos: An information aesthetic for comparative genomics. Genome Res..

[106] Kayum M.A., Park J.I., Ahmed N.U., Jung H.J., Saha G., Kang J.G., Nou I.S. (2015). Characterization and stress-induced expression analysis of Alfin-like transcription factors in Brassica rapa. Mol. Genet. Genom..

[107] Reich M., Liefeld T., Gould J., Lerner J., Tamayo P., Mesirov J.P. (2006). GenePattern 2.0. Nat. Genet..

[108] AtGenExpress Visualization Tool (AVT). http://jsp.weigelworld.org/expviz/expviz.jsp.

[109] Cluster Program. http://bonsai.hgc.jp/~mdehoon/software/cluster/software.htm.

[110] GenePattern Software. http://genepattern.broadinstitute.org/gp/pages/index.jsf.

[111] NCBI Database. http://www.ncbi.nlm.nih.gov/.

