# Supplementary material for: Glutathione Transferases Superfamily: Cold-Inducible Expression of Distinct GST Genes in Brassica oleracea
Source: Int J Mol Sci. 2016 Jul 27;17(8):1211. doi: 10.3390/ijms17081211 (PMC5000609; doi:10.3390/ijms17081211)
Supplement: Supplementary file 1 [file ijms-17-01211-s001.zip › ijms-137475-Supplementary Materials/ijms-137475-Supplementary figures.pdf]

Int. J. Mol. Sci. **2016**, *17*, 1211; doi:10.3390/ijms17081211

Supplementary Materials: Glutathione Transferases Superfamily: Cold-Inducible Expression of Distinct *GST* Genes in *Brassica oleracea*

Harshavardhanan Vijayakumar, Senthil Kumar Thamilarasan, Ashokraj Shanmugam, Sathishkumar Natarajan, Hee-Jeong Jung, Jong-In Park, HyeRan Kim, Mi-Young Chung and Ill-Sup Nou

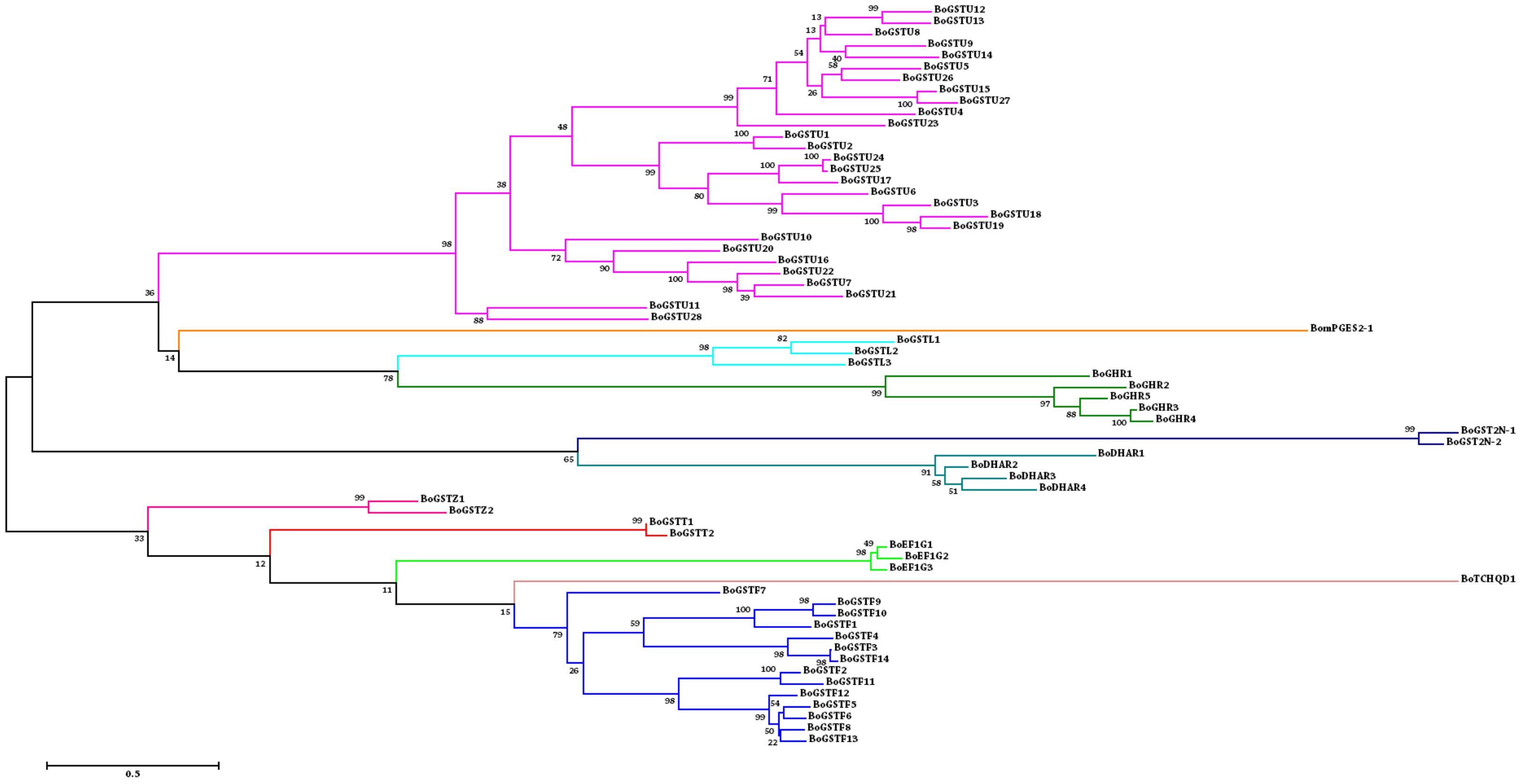

Figure S1. Phylogenetic relationship of BoGST proteins in *Brassica oleracea*.

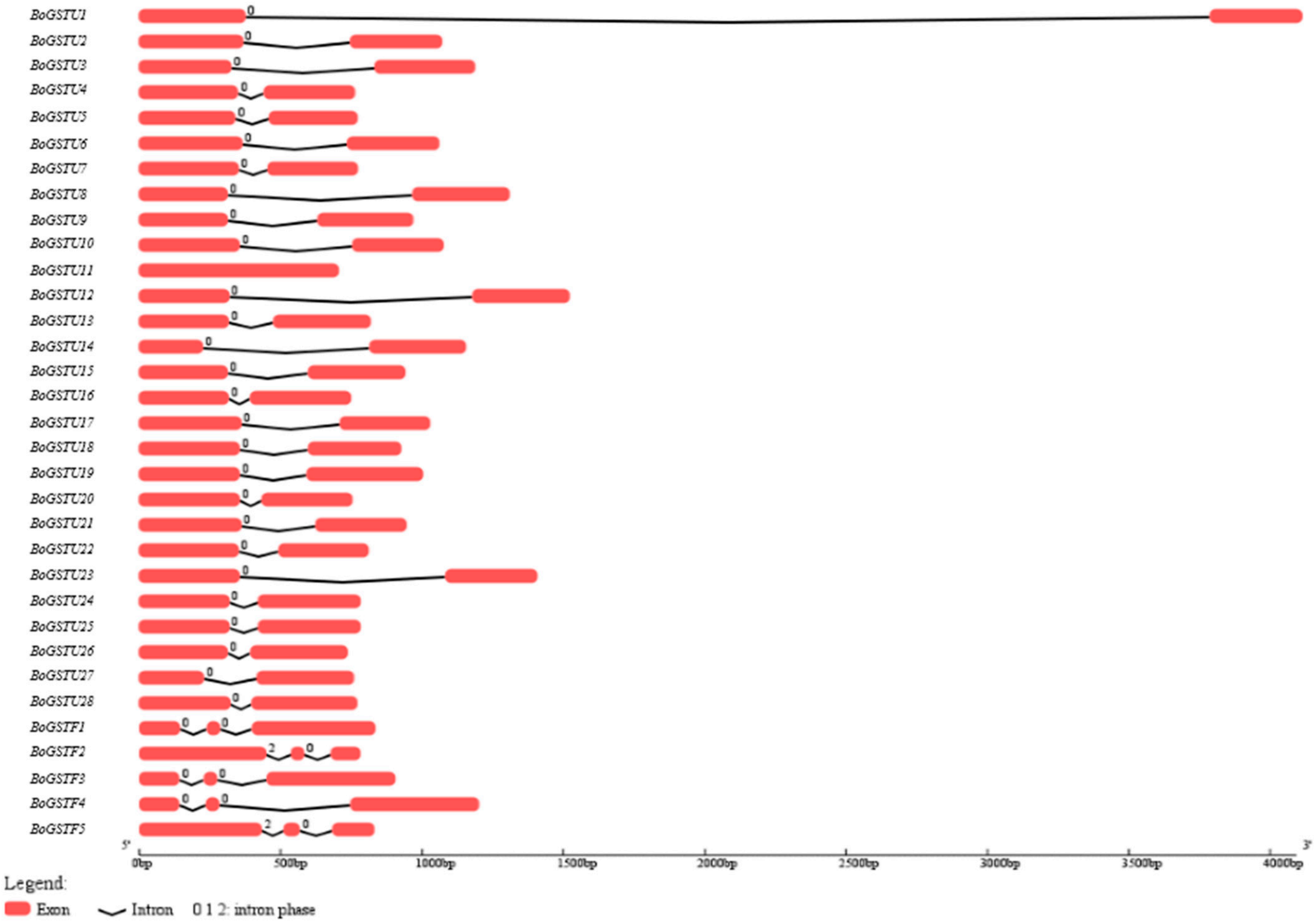

(**a**)

Figure S2. *Cont.*

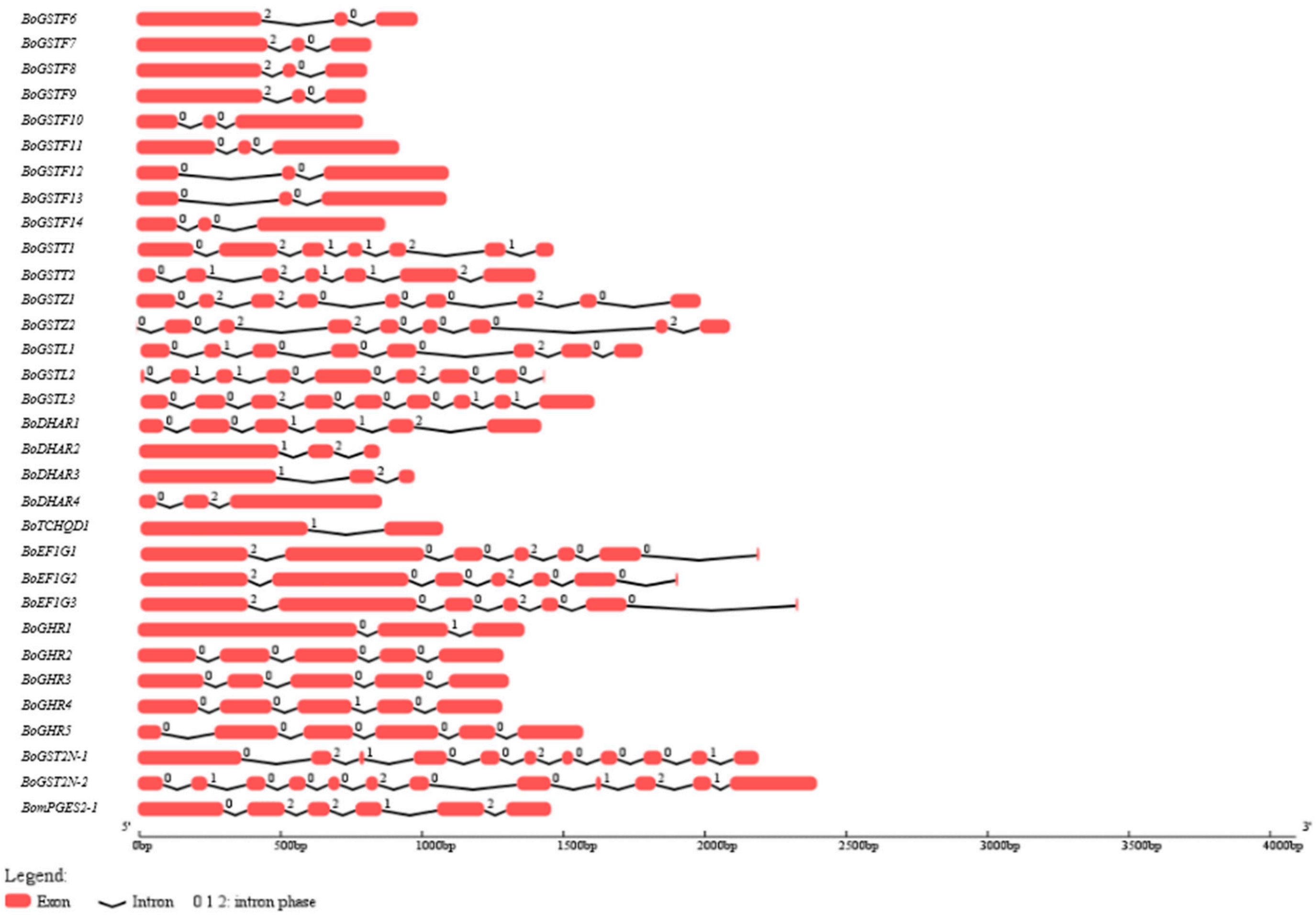

(**b**)

Figure S2. Intron-exon structure of *BoGST* genes. Red bar indicates exon, black line indicates intron position and the number on intron represent intron phase (0-between two consecutive codons, 1-between the first and second nucleotide of a codon, 2-between the second and third nucleotide of a codon).

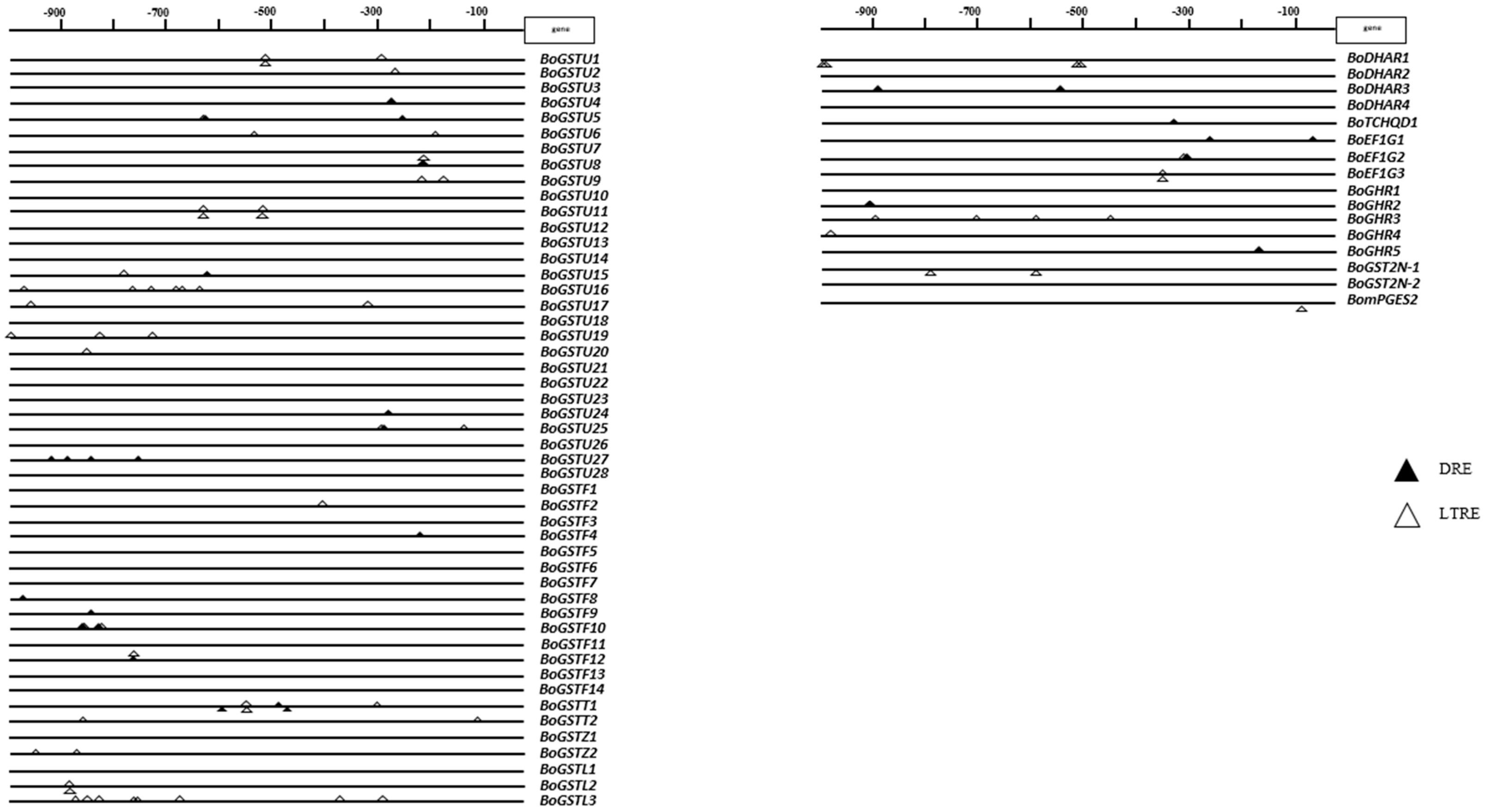

Figure S3. Putative DRE, and LTRE elements were predicted promoter regions (1000 bp) of the cold-inducible BoGST genes. The lines represent promoter sequences. The elements located in the forward strand (sense strand) and the reverse strand (antisense strand), were indicated below and above the lines, respectively.

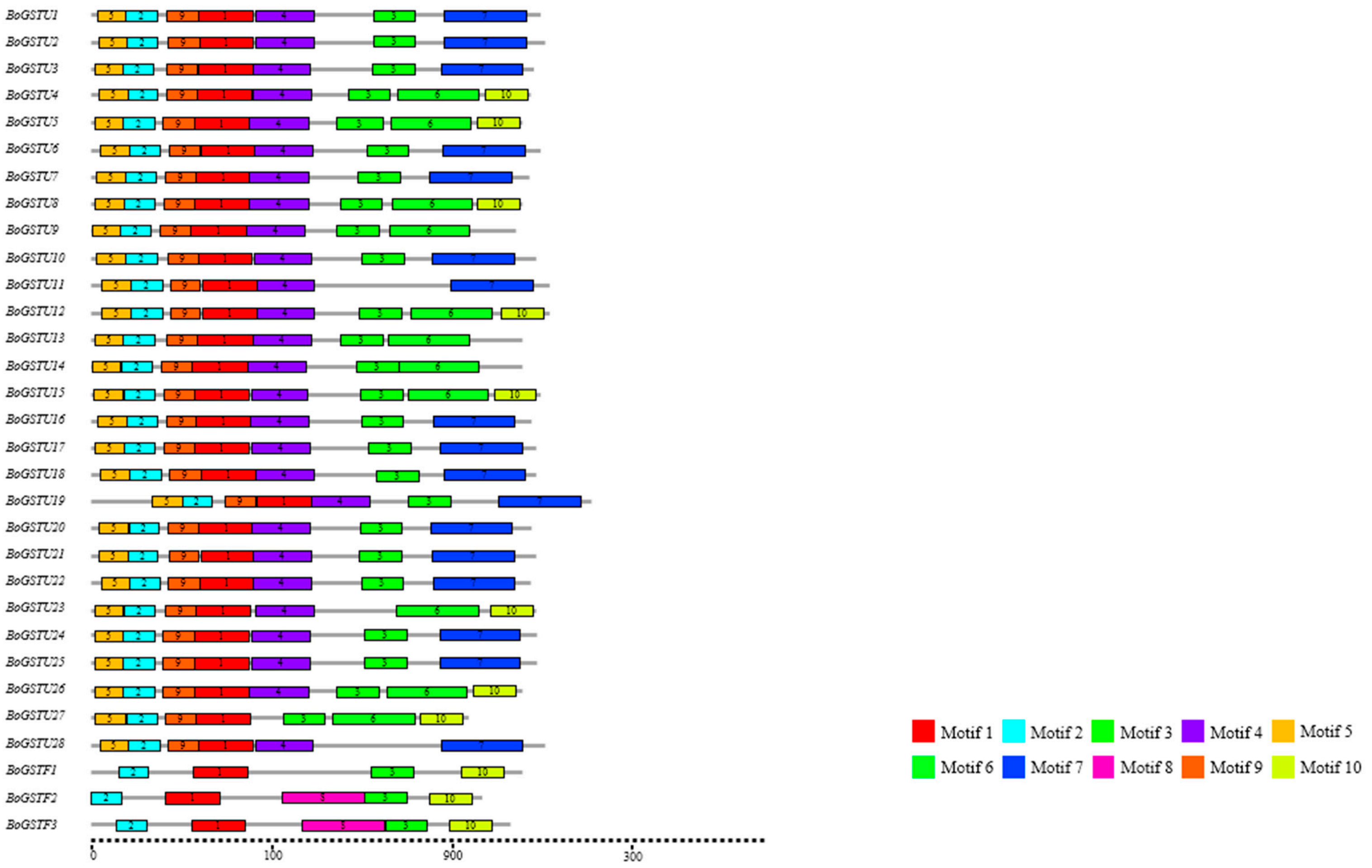

(**a**)

Figure S4. *Cont.*

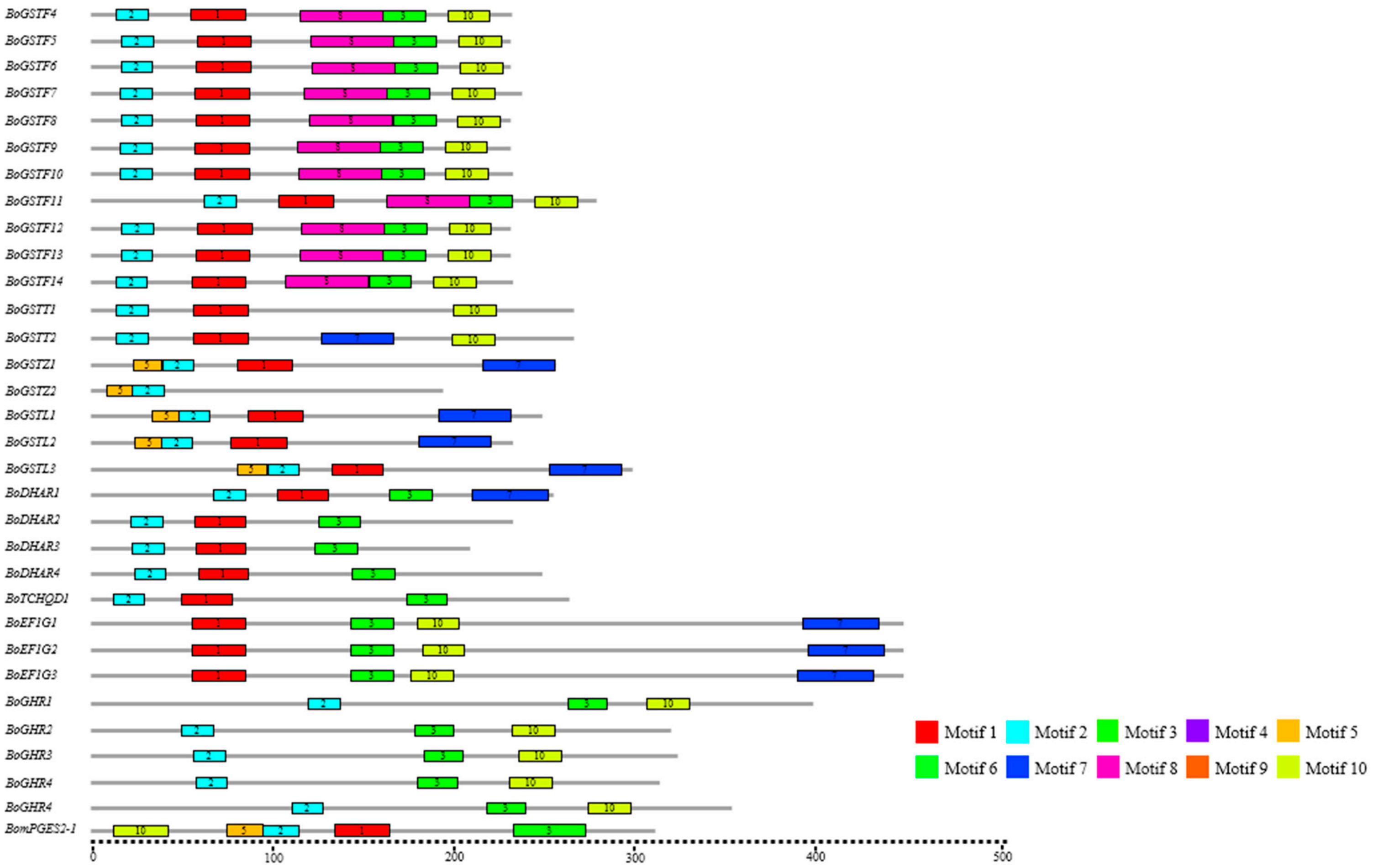

(**b**)

Figure S4. Schematic representation of motifs organization identified in BoGST proteins. Different motifs are indicated by different colors and the names are mentioned on the left side.

Motifs	Consensus Sequence	Length (aa)	No. of GST proteins	E-value
1	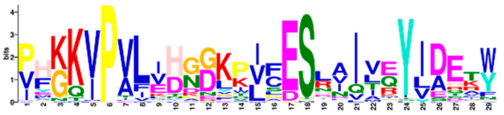	29	57	6.4e-750
2	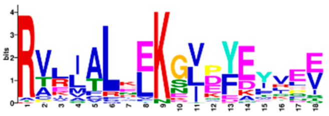	18	59	3.4e-313
3	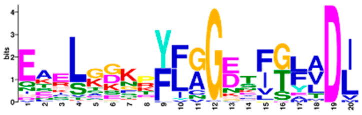	20	53	9.6e-327
4	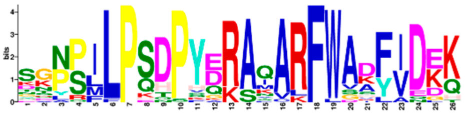	26	26	4.6e-332
5	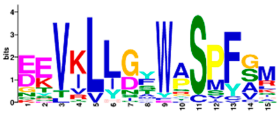	15	33	1.7e-152
6	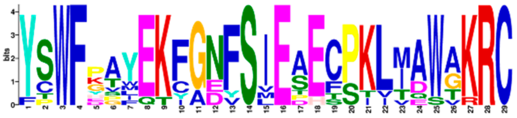	29	11	2.10E-150
7	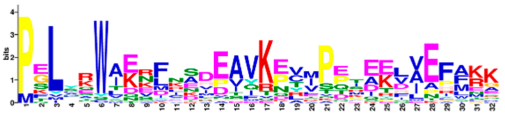	32	26	2.6e-120
8	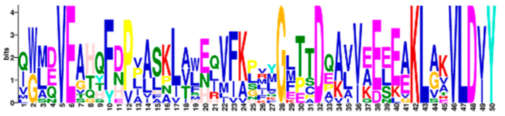	50	13	5.3e-220
9	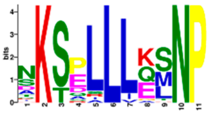	11	27	5.2e-106
10	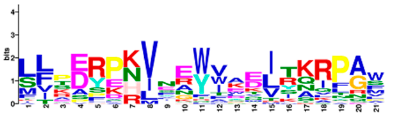	21	34	1.3e-102

Figure S5. Putative motifs predicted in BoGST proteins. Significant motifs (e-value < e-100) GST proteins were predicted by MEME search. The consensus sequence, length (amino acids), number of GST proteins containing the motif and e-value of each predicted motif is given. The 18th residue in motif 1 represents the active site serine residue.

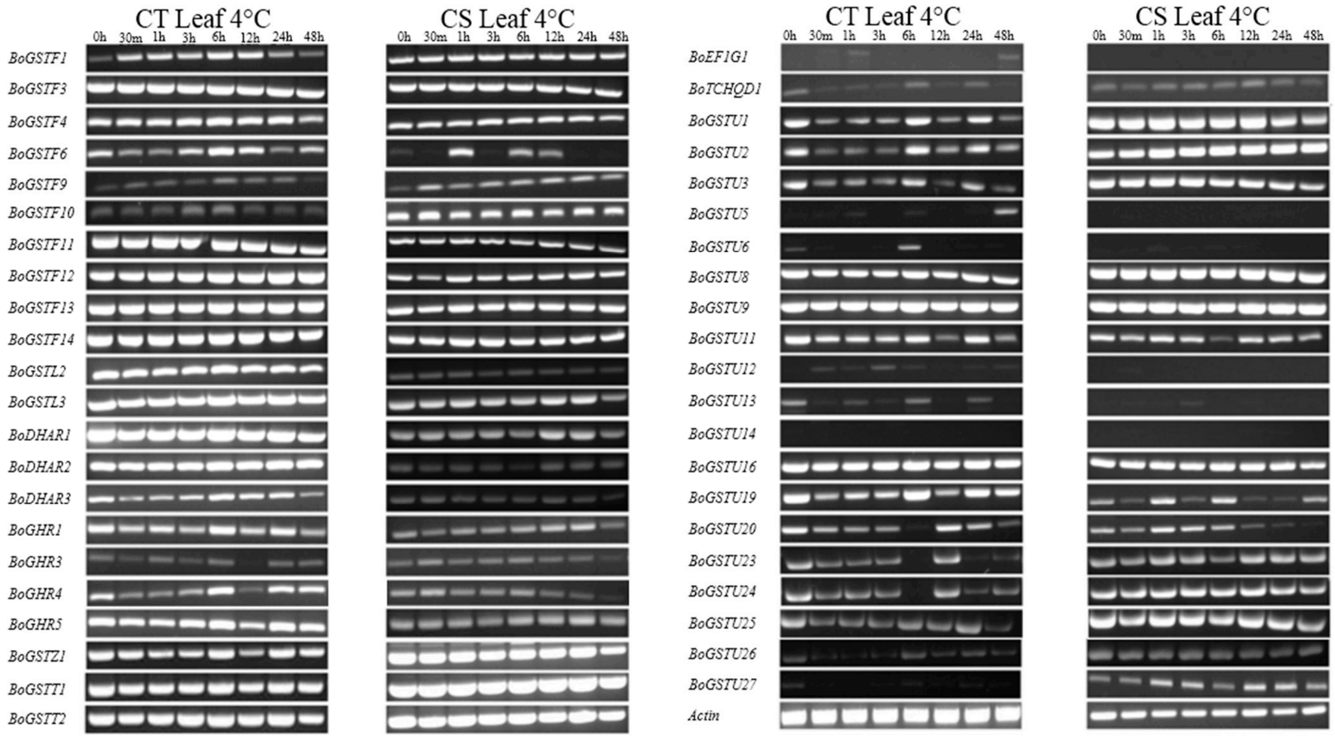

Figure S6. RT-PCR analysis of 41 BoGST genes expressed in two contrasting cold genotypes (Bo106 and Bo107) during cold stress in leaf samples. Actin was used as housekeeping gene.
